# Chronic pruritus: a narrative review^☆^

**DOI:** 10.1016/j.abd.2024.09.008

**Published:** 2025-05-03

**Authors:** Paulo Ricardo Criado, Roberta Fachini Jardim Criado, Mayra Ianhez, Hélio Amante Miot

**Affiliations:** a Centro Universitário Faculdade de Medicina do ABC, Santo André, SP, Brazil; b Faculdade de Ciências Médicas de Santos (Fundação Lusíada), Santos, SP, Brazil; c Alergoskin Alergia e Dermatologia, UCARE Center and ADCARE, Santo André, São Paulo, Brazil; d Department of Dermatology, Hospital de Doenças Tropicais de Goiás (HDT-GO), Goiânia, GO, Brazil; e Department of Infectology, Dermatology, Imaging Diagnosis and Radiotherapy, Faculty of Medicine, Universidade Estadual Paulista, Botucatu, SP, Brazil

**Keywords:** Dermatitis atopic, Dupilumab, Histamine antagonists, Janus kinase inhibitors, Phototherapy, Prurigo, Prurigo nodularis, Pruritus

## Abstract

Chronic pruritus encompasses a manifestation of several cutaneous, allergic, infectious, neurological, psychological, and systemic conditions, whose etiological investigation and therapeutic strategy can be challenging. This comprehensive review aims to enhance the understanding of pruritus by highlighting important elements in its pathogenesis, including keratinocytes, Merkel cells and mast cells, nerve fibers, histaminergic and nonhistaminergic pathways, and the interaction of itch signals with the central nervous system. Diagnostic evaluation of chronic pruritus may require a meticulous approach, guided by the identification of skin lesions or signs/symptoms of underlying systemic diseases. A comprehensive evaluation, including a detailed medical history, thorough physical examination, and appropriate laboratory and imaging tests, often supplemented by skin biopsy and direct immunofluorescence, is essential. Treatment strategies for chronic pruritus should be individualized based on the etiology identified. General measures, such as emollients, serve as initial interventions, followed by targeted approaches. Topical corticosteroids, calcineurin inhibitors, phototherapy, and systemic immunosuppressants address cutaneous inflammation. Antihistamines, antidepressants, and immunosuppressants may be employed based on the specific etiology. Emerging therapies, including biologic drugs and JAK inhibitors, have potential in refractory cases.

## Introduction

Pruritus (itching) is the most frequently reported symptom among patients who see dermatologists.^1^ It was defined by the German physician Samuel Hafenreffer more than 350 years ago as an “unpleasant sensation that provokes the urge or reflex to scratch”.^2^, ^3^

Chronic Pruritus (CP), i.e., pruritus that lasts more than 6 weeks,^1^ has an estimated prevalence ranging from 8% to 25% and can be localized or generalized.^4^, ^5^, ^6^ The prevalence of CP in children aged 6 to 10 years is estimated at 15%^7^ and in older people (≥65-years), it is 25%.^8^

Patients suffering from CP often experience a significant impact on psychosocial well-being, including sleep disturbances, shame, or even body dysmorphic disorders due to visible injuries caused by scratching.^9^, ^10^, ^11^ Patients with severe itching have a lower quality of life and suffer more from depressed mood and anxiety. Suicidal ideations were reported in 18.5% of patients with CP.^12^

The International Forum for the Study of Itch (IFSI) has classified chronic pruritus into three categories: (i) Chronic Pruritus on primarily Lesional (altered) skin (CPL), where an underlying skin disorder is present; (ii) Chronic Pruritus on Primarily Non-Lesional (unchanged) skin (CPNL), where there are no initial skin lesions (formerly known as pruritus sine materia); and (iii) Chronic pruritus with severe scratching lesions (e.g., chronic prurigo, lichen simplex), which prevents classification into the first or second category.^13^ This classification is crucial for guiding both diagnosis and treatment, as the underlying mechanisms and therapeutic strategies may vary significantly depending on the type of pruritus.

The primary aim of this review is to explore the pathogenesis of CP, its origins in the skin/mucosa or Central Nervous System (CNS), with particular attention to these different distinctions of pruritus and the causes related to underlying dermatological conditions, internal diseases, or when classified as Chronic Pruritus of Unknown Origin (CPUO), along with current recommendations for investigation and treatment.

## Pathogenesis of chronic pruritus

Histamine was the first mediator identified in association with pruritus. However, antihistamine therapy has proven effective only in treating a few conditions, such as hives, allergic drug reactions, and insect bite reactions. Pruritus is a symptom resulting from a complex interaction of inflammatory mediators, immune cells, skin cells, and neuronal networks, involving both the peripheral and central nervous systems to produce the characteristic scratching response. The process begins in the epidermis and dermal-epidermal junction, where a pruritogen ‒ originating from immune cell products, exogenous compounds, or keratinocytes ‒ activates pruritic receptors on unmyelinated C-nerve fibers.^14^ These fibers can be classified as histaminergic or non-histaminergic based on receptor expression.^15^ Histaminergic nerve fibers are typically involved in the transition from acute itch to histamine-activated pruritus, whereas Chronic Pruritus (CP) is associated with non-histaminergic fibers, which are activated by pruritogens other than histamine.^16^

**Fig. 1:**
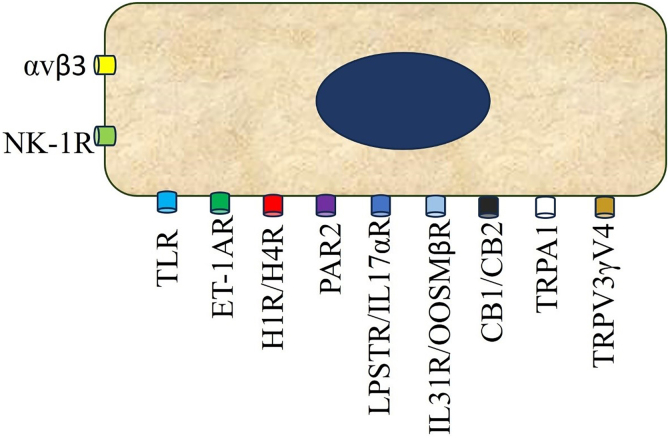
Keratinocyte receptors enrolled in pruritus.

**Fig. 2:**
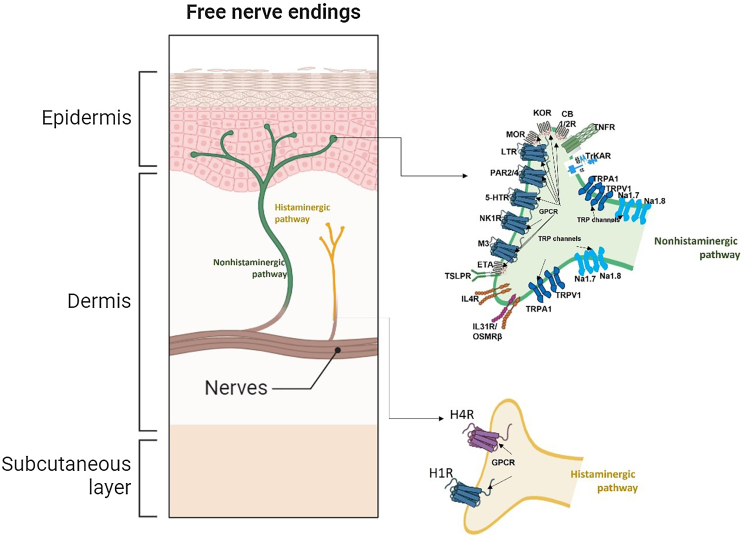
Pruritus receptors (PNS) in the epidermis and dermis. There are at least 3 subsets of pruriceptors expressing dedicated pruritogen receptors. These neurons are generally subdivided into NP1, NP2, and NP3 neurons based on their expression patterns. Cation channels transient receptor potential vanilloid subtype 1 and/or transient receptor potential ankyrin1 and NaV1.7 and NaV1.8, respectively, are required for downstream signaling of the itch receptors and are consistently expressed across subsets of these neurons (nonhistaminergic pathway). Histaminergic itch results from the direct action of the pruritogen histamine on its receptor (H1R) and (H4R) on sensory nerves to transmit itch signals. Although the H1R is coexpressed with other pruritogen receptors on pruriceptors, its utility as an itch therapeutic target is limited to certain inflammatory itch diseases as such as urticaria. Abbreviations: 5-HTR, serotonin receptor; CB1/2, cannabinoid receptor type 1 and 2; ETA, endothelin A receptor; H1/4R, histamine receptor type 1 and 4; IL31R, interleukin 31 receptor; IL4R, interleukin 4 receptor; KOR, kappa opioid receptor; LTR, leukotrienes receptor; M3, muscarinic acetylcholine receptor 3; MOR, mu opioid receptor; Nav1.7/1.8,voltage-gated sodium channel; NK1R, neurokinin 1 receptor; OSMRβ, oncostatin M receptor beta; P, phosphate; PAR2/4, protease-activated receptor type 2 and 4; TNFR, tumor necrosis factor receptor; TrkA, tropomyosin receptor kinase A; TRPA1, transient receptor ankyrin 1; TRPV1, transient receptor potential vanilloid 1;TSLPR, thymic stromal lymphopoietin receptor. Image partially created using BioRender®.

Dysregulated communications between sensory nerve endings, immune cells, keratinocytes, skin-resident cells, as well as the CNS trigger CP chronification. After triggering cutaneous stimuli, itching signals are sent to the peripheral nerve from cutaneous nerve endings, which originate from the Dorsal Root Ganglion (DRG), ascend to the somatosensory thalamus, and are then projected into the cerebral cortex (Fig. 1, Fig. 2).^17^

Itchy skin involves the main components: (i) Skin-resident cells; (ii) Itching nerve fibers (PNS); (iii) Itch receptors; (iv) Histaminergic and nonhistaminergic itch pathways; (v) Transmission of itching in the spinal cord; (vi) Itching in the brain (CNS).^18^

(i) Resident cells in the skin

### Keratinocytes

They are considered to be on the front line of the nervous system, given their high bioactivity and ability to secrete pruritogens.^19^ They express Protease-Activated Receptor (PAR2), Toll Receptor 3 (TRL3), Histamine Receptors (HR1-HR4), Endothelin A (ETA) and B (ETB), serotonin (5-HTR), Oncostatin M β receptor (OSMRβ), integrin αvβ3, Thymic Stromal Lymphopoietin Receptor (TSLPR), Neuropeptide Y receptors (NPY), including TRPV3 and V4 (Fig. 1)

The indirect pathway of itch induction involves the disruption of the epidermal barrier, leading to an increased inflammatory gradient and transepidermal water loss. This disruption is typically accompanied by the production of pro-inflammatory cytokines (e.g., IL-6) and various chemokines (e.g., CXCL-8, CCL17/TARC, CCL19/MIP-3β, CCL22/MDC, CCL23/MIP-3, CCL4/MIP-1β, and CXCL1/GRO1α), as well as Nerve Growth Factor (NGF) by keratinocytes. The main pruritic mediators released by keratinocytes that can directly activate pruritic nerve endings include TSLP, periostin, ET-1, IL-33, and BNP.^19^, ^20^

TSLP and other Th2 cytokines may induce periostin secretion, which in turn can stimulate further TSLP release, potentially establishing a pruritic positive feedback loop. One of the most potent pruritic mediators derived from keratinocytes is ET-1, whose production can be triggered by the activation of PAR2, TLR3, TRPV3, and TRPV4. IL-33, a member of the IL-1 family of inflammatory cytokines, is constitutively expressed in the nucleus of keratinocytes and acts as an alarmin, released in response to inflammation or cell damage.^20^

Initially, IL-33 was shown to act on cells of both the innate and adaptive immune systems, specifically mast cells, Innate Lymphoid Cells type-2 (ILC2), basophils, and type-2 helper T-cells. However, its receptor, ST2, is also expressed on sensory nerve endings in the skin, and its activation leads to an itch response in mice. Additionally, IL-33 is upregulated in Atopic Dermatitis (AD) lesions, potentially contributing to the pruritic phenotype associated with this condition.^20^

### Merkel cells

These cells trigger the pruritus-scratch cycle after stimulation by the Piezzo receptor-2.^19^ The complex formed by Merkel cells and pruriceptive MRGPRA3+ endings (C fibers) in cases of xerosis and prurigo nodularis may be functionally impaired. Merkel cells express TRPM8.^19^

### Mast cells

These innate immune cells are located in the papillary dermis, near nerve fibers, around the pilosebaceous unit, around dermal blood vessels, and are loaded with pruritogens, capable of activating both histaminergic and non-histaminergic pathways.^19^

(ii) Itch nerve fibers

The cell bodies of the myelinated Alpha-Delta (Aδ) fibers (mechanical itch), unmyelinated C-fibers (CM and CMH), both non-histaminergic, and the unmyelinated C-fibers (CMi/histaminergic) reside in the Dorsal Root Ganglia (DRG) with axons innervating the skin (epidermis and dermis), and dendrites synapsing in the dorsal horn of the spinal cord.^18^ Pruritus is initiated when endogenous and exogenous pruritogens bind to their receptors on these sensory nerve endings.^18^

(iii) Itch receptors and channels^18^, ^21^

There are 3 major classes of *itch receptors*: G Protein-coupled Receptors (GRPs), Toll-Like Receptors (TLRs) and cytokine receptors (for example, interleukin-31, thymic stromal lymphopoietin, interleukin-4, interleukin-13, interleukin-33, Omcostatin-M [OSM]).^18^, ^21^ The members of the Mas-related GPCR (MRGPR) involved on itch are represented by MRGPRX1 and MRGPRX4 in humans, and as an example, chloroquine elicits itching through MRGPRA3 stimulation in rodents and MRGPRX1 in humans.^21^ One class of channels broadly associated with pruritus is the Transient Receptor Potential (TRP) channels. This group includes TRP Vanilloid-1 (TRPV1) and TRP Ankyrin-1 (TRPA1), which activate the Nav1.7 and Nav1.8 sodium channels, thereby propagating the action potential of the itch signal. Both histaminergic and non-histaminergic itch pathways rely on TRP signaling. Table 1 provides a summary of the distinct itch receptors, their activating compounds, and the associated pathways or diseases.^22^, ^23^

**Table 1:** Itch channels, receptors, their activating compounds and associated pathways, conditions or diseases.^20^, ^21^, ^22^, ^23^, ^24^, ^25^.

Channels / Receptors	Type	Activating Compounds	Associated Pathways/Diseases	
*Transient receptor potential (TRP)* channels	TRPA1 (TRP Ankyrin 1)	Allicin (found in garlic), allyl isothiocyanate (alkaloid found in mustard oil, wasabi, and horseradish), arachidonic acid, BAM8-22, bile acids, bradykinin, carvacol, chroloquine, cowhage, cinnamaldeyde (found in cinnamon oil), endothelin-1, hydrogen peroxide, IL-33, IL-31, LPA, LTB4, prostaglandins, t-BHP, TSLP, 5-HT (serotonin), periostin	*MRGPR-associated nonhistaminergic pruritus and PAR-mediated nonhistaminergic pruritus*; conditions as AD, allergic contact dermatitis, cholestatic pruritus, psoriasis	
	TRPV1 (TRP Vanilloid 1)	Capsaicin, histamine, ATP, lipoxygenase products, prostaglandins, imiquimod, periostin, IL31, IL-33	*Histaminergic pruritus and PAR-mediated nonhistaminergic pruritus*, *IL-31 and IL-33 mediated itch pathways*; conditions: AD, psoriasis, prurigo nodularis.	
	TRPV2 (TRP Vanilloid 2)	Increased temperature, physical stimuli	Mast cell degranulation, PKA-mediated inflammatory cascade	
	TRPV3 (TRP Vanilloid 3)	Plant-derived compounds, arachidonic acid, farnesyl pyrophosphate (FPP)	*PAR-mediated nonhistaminergic pruritus*, IL-31- mediated BNP synthesis; Conditions: Olmsted syndrome (missense mutation with gain-of-function of the gene codifying TRPV3), AD, psoriasis	
	TRPV4 (TRP Vanilloid 4)	Histamine, endothelin-1, 5-HT, lysophosphatidylcholine (LPC)	*Histaminergic pruritus*; dry skin pruritus, allergic contact dermatitis, psoriasis, chronic idiopathic pruritus	
	TRPM8 (TRP Melastatin 8)	Menthol, icilin	*Histaminergic and nonhistaminergic pruritus*; B5-1 neuron-associated spinal interneuron circuit; dry skin pruritus, AD, urticaria, scalp pruritus	
	TRPC3 (TRP Cannonical 3)	Chloroquine, beta-alanine	*Nonhistaminergic pruritus*; contact dermatitis	
	TRPC4 (TRP Cannonical 4)	Sertraline	Sertraline receptor HT2B-associated pruritus	
*G protein-coupled receptors (GRPs)*	Receptors (human and murine)		Ligand endogen	Ligand exogen
	MRGPX2 (human ortholog), MrgprB2 (murine ortholog)		Substance P, platelet factor-4, AC-30/SC, β-defensin, cathelicidin, LL-37, BAM (8‒22; 13‒22; 22), catestatin, cortistatin, hemokinin-1	Ciprofloxacin, Levofloxacin, moxifloxacin, ofloxacin, atracurium, rocuronium, tubocurarine, angiopeptin, cetrorelix, hexarelin, icatibant, leuprolide, octreotide, sermorelin, compound 48/80, mastoparan, CSP-CSP1-CSP-2, entf, streptin-1
	MRGPRX1 (human ortholog), Mrgprc11 (murine ortholog)		BAMB98-220, β-defensin, SLIGL [Tyr^6^] γ2-MSH96-12), neuropeptide FF	IPDef1; IRDef2
	MRGPRX1 (human ortholog), Mrgpra3 (murine ortholog)		β-defensin	Chroroquine
	MRGPRX1 (human ortholog), Mrgpd (murine ortholog)		β-alanina, alamandine, 5-oxoETE, angiotensin (1‒7), GABA	ND
	MRGPRX4 (human ortholog), Mrgpra1 (murine ortholog)		Bilirubin, Salusin β, Arg-Phe-amide containing neuropeptides (FLRF-amide, FMRF-amide and NPFF)	ND
	Mrgpra4		Neuropeptide FF, ACTH	ND
*Toll-like receptors (TLRs)*^22^, ^23^	TLRs function as innate sensors in the immune system.^22^ They may have a similar role in the nervous system, but this possibility has not been demonstrated conclusively.^22^ Chronic itch after skin injury also requires TRPA1. Several types of nociceptor-expressed TLRs, such as TLR2, TLR3, TLR4, and TLR7, have been implicated in itch modulation via functional coupling to TRPA1 or/and TRPV1.^23^		TLR3, TLR7, and potentially TLR4 are expressed on small-sized primary sensory neurons. TLR3, TLR7, and potentially TLR4 are expressed in a subtype of pruriceptive/nociceptive neurons in the dorsal root and trigeminal ganglion providing a direct link between TLR activation and itch.^22^ Activation of neuronal TLRs can initiate itch sensation by coupling with ion channels.^22^ Furthermore, TLRs are expressed in skin cells and glial cells in the spinal cord to regulate inflammation and neuroinflammation in chronic itch.^22^	
*Cytokine Receptors*^24^	TSLP and its receptor complex IL7Rα/TSLPR^24^, ^25^ The intracellular signalization of these interleukin receptors is mediated by TRPA1.		Expressed by a small subset of itch-sensing DRG neurons.^24^ High TSLP skin expression is a hallmark feature of AD, and TSLP is released by KCs in response to a broad range of stimuli, including in allergy and proteolytic PAR2 activation.^24^	
	IL-33 and their receptor complex ST2/IL1RAP^24^, ^25^ The intracellular signalization of these interleukin receptors is mediated by the channels TRPA1/TRPV1.		Expressed by a subset of histamine-sensitive DRG neurons.^24^ However, the exact involvement of IL-33 in itch is less clear.^24^ Although this cytokine is important for the development of chronic itch conditions such as ACD and xerosis.^24^ IL-33 has been reported to stimulate enkephalin production in group 2 innate lymphoid cells.^24^ The derivative of proenkephalin A-bovine adrenal medulla 8–22 is a potent MRGPR agonist.^24^	
	Type-2 immune cell-derived Interleukins (ILs)- IL-4, IL-13, IL-31 and their receptors^24^, ^25^ The intracellular signalization of IL-13Rα1 and IL-4Rα are mediated by JAK1/STAT pathway.		These type-2 cytokines lay a major role in AD-associated itch.^24^ The involvement of these cytokines in AD skin disease had already been known, but these cytokines have recently also been implicated in the direct modulation of itch neurons.^24^ In mice, the IL-4 and IL-13 receptor complex IL-4Rα/IL-13Rα1 is broadly expressed by itch-sensing DRG neurons, and the IL-31 receptor complex IL-31Rα/OSMRβ is further expressed by some 5-HT – sensitive fractions of these neurons.^24^ The expression of these receptors has also been detected in human DRGs.^24^ These cytokines broadly enhance itch neuronal excitability, thereby potentiating both histaminergic and nonhistaminergic itch pathways.^24^	

ACTH, Adrenocorticotropic Hormone; AD, Atopic Dermatitis; BAM, Bovine Adrenal Medulla; BAM, Bovine Adrenal Medulla; BNP, B-type Natriuretic Peptide; CSP, Competence-Stimulating Peptide-1; GABA, Gamma Aminobutyric Acid, IL, Interleukin; IPDef1 (IP defensin-1) and IRDef2 (IR defensin-2), both are tick salivary peptides; JAK/STAT, Janus Kinase/Signal-Transducer and Activator of Transcription; LTB4, Leukotriene B4; LPA, Lysophosphatidic Acid; 5-HT, 5-Hydroxyptamine (serotonin); oxoETE, 5-Oxieicosatetraenoic acid; LPA, ND, Not Determined; MSH, Melanocyte-Stimulating Hormone; NPFF, Neuropeptide FF; PAMAP, Proadrenomedullin Peptide, PACAP, Pituitary Adenylate Cyclase Activating Polypeptide; PAR, Protease Activated Receptor; PKA, Protein Kinase-A; VIP, Vasointestinal Peptide, TSLP, Thymic Stromal Lymphopoietin.

There are several chemical stimuli that trigger itch at different stages, including neuropeptides, amines, cytokines, chemokines, proteases, lipids, and opioids and their respective receptors, as demonstrated in Table 2.^24^ Mediator related itch implies that pruritus is associated with mediators including histamine, 5-hydroxy tryptamine, proteases, opioid peptides, peptides, and eicosanoids.^24^

(iv) Histaminergic and nonhistaminergic pathways

**Table 2:** Distinct mediators and receptors involved on chronic pruritus.^20^, ^25^, ^26^, ^27^

Classes of mediators and/or targets involved on pathogenesis of itch	Mediators	Cells involved, actions and disorders associated
Neuropeptides (critical in the transmission of itch sensation from the peripheral nervous system to the spinal nervous system and to brain)	*Calcitonin gene-related peptide (CGRP)*	Vasodilator agent expressed in the sensory neurons, motor neurons, monocytes, macrophages, Langerhans Cells (LCs) and keratinocytes. CGPRα is expressed in the CNS and Peripheral NervousS (PNS), whereas CGRPβ is less found in PNS, but is the only form found in keratinocytes.^20^ The CGRP is released after activation of *transient receptor potential cation channel subfamily V member 1* (TRPV1, also known as the capsaicin receptor and the vanilloid receptor-1) on membrane of sensory neurons.^20^ CGRP act on several immune cells, including-T and B-cells, Dendritic Cells (DC), mast cells, macrophages, LCs, causing neuro-inflammation, neurogenic vasodilatation, and immune response. CGRP^+^ interneurons (in DRG) may mediate spinal itch transmission, but not pain signals.^20^
	*Substance P (SP)*	SP is an important transmitter of the afferent neurons in the PNS and CNS.^25^ SP acts in distinct immune cells, such as eosinophils, mast cells, T-cells and promotes skin inflammation. *Neurokinin-1 receptor* (NK-1R) is traditionally regarded as the main SP receptor, however on mast cells*, mas-related G-protein-coupled receptor member B2* (MrgprB2/MrgprX2) may be the critical receptor for SP-mediated mast cell activation. SP actives mast cells to release histamine, Leukotriene B4 (LTB4), Prostaglandin D2 (PGD2), and Tumor Necrosis Factor alpha (TNF-α).^25^ These mediators reach sensory nerve endings in epidermis and dermis where induce SP release and exacerbate itch, in a vicious cycle of positive feedback for itch.^25^ SP released from the sensory nerve endings, especially from C-fibers, can increase histamine and TNFα release from mast cells, IL-1, IL-6 and IL-8 production in keratinocytes, or IL-8 production in dermal microvascular endothelial cells, all contributing to local inflammation.^25^
	*Neuropeptide Y (NPY)*	Spinal inhibitory interneurons express receptors for this molecule which form synapses with afferent fibers stimulated by secretion of natriuretic peptide B.^27^
	*Neurotrophin*	Neurotrophin is a large family of physiological activators promoting the growth, differentiation, and maintenance of neurons.^26^ It primarily contains Nerve Growth Factor (NGF), Brain-Derived Neurotrophic Factor (BDNF), Neurotrophic Factors-3 (NT-3), and Neurotrophic Factors-4 (NT-4).^26^ NGF levels in the itchy lesions of AD and psoriasis significantly increased and correlated with the severity of diseases;^26^ NGF, at the same time, upregulated the expression of sensory neuropeptides, which may induce the release of TRPV1, elicit the degranulation of mast cells, and result in pruritus.^26^
	*B-type natriuretic peptide (BNP)*	BNP is a central itch mediator.^5^ Release and synthesis of BNP is upregulated by Interleukin-31 (IL-31) in sensory Dorsal Root Ganglionic Neurons (DRGs).^20^ IL-31 receptors (IL-31RA and OSMR) are co-enriched with the BNP gene (Nppb) in DRG.^20^ BNP and receptor expression is increased in the pathogenic skin of AD patients.^20^ In the human skin cells, the pro-inflammatory and itch-promoting phenotypes are promoted by BNP.^20^ In the skin, BNP was found to sensitize Transient Receptor Potential Vanilloid-3 (TRPV3), resulting in enhanced Serpin E1 release, an itch-specific mediator with transcription levels positively correlated with the severity of human AD skin.^20^
	*Gastrin-releasing peptide (GRP)*	GRP is a spinal itch-selective transmitter known to be highly expressed in a population of spinal cord Dorsal Horn (DH) interneurons and may serve as a “leaky gate” for nociceptive signals.^20^ GRP neurons receive direct input from MrgprA3+ pruritoceptors.^20^ GRP level is also increased in AD patient skin and GRP seems to promote Thymic Stromal Lymphopoietin (TSLP) release from keratinocytes.^20^
	*Somatostatin (SST)*	SST originates from the DRGs and spinal dorsal horn neurons and is an important endocrine hormone and a neuropeptide in the nervous system, including the PNS and CNS.^20^
	*Opioid peptides*	Opioid peptides have peripheral and central itchy effects. They effectively work by activation of µ-Receptor (MOR) and inhibition of κ-Receptor (KOR) in the central nervous system. MOR is the major functional receptor for itching production, but KOR does the opposite. At the periphery, on the other side, morphine induces pruritus generation by eliciting the degranulation of mast cells.^26^ All of these opioid peptides could cause itching after intrathecal administration.^26^ Endogenous endorphins demonstrate affinity for MOR expressed in C and A fibers.^27^ These form synapses with GRPR + excitatory interneurons in the posterior horn, where signaling is pruritogenic.^27^ Th e KOR bind to dynorphin, whose activity is antipruritogenic.^27^ At physiological level there is a homeostasis between MOR and KOR activation, controlled mainly by excretion of dynorphin.^27^ In the spinal cord, there is a subpopulation of dynorphin + inhibitor interneurons.^27^ Loss of function of these interneurons are implicated in central signaling of pruritus and alloknesis.^27^
Amines	*Histamine*^27^	Histamine is a chemical medium mainly stored in the basophilic leukocyte and mast cells.^26^ When these cells are activated by immune and nonimmune factors, histamine is induced to release.^26^ Its receptors belong to the members of the G Protein-Coupled Receptors (GPCR), in which H1 and H4 Receptors (H1R and H4R) play important roles in the appearance of pruritus.^26^ Previously, it was considered that histamine dominated the development of pruritus via binding to H1R and activating phospholipase Cβ3 (PLCβ3) and Phospholipase A2 (PLA2).^26^ HR-4 is present in keratinocytes and in C fibers (histaminergic).^27^ Histamine could increase the calcium influx in the axon terminals of the spinal cord neurons by activating Transient Receptor Vanilloid-1 (TRPV1) receptor and then promote a series of intracellular signal activation and ultimately lead to itching generation.^26^ Nowadays, histamine is the main mediator in some conditions as acute and chronic urticaria, a few drugs adverse reactions and insect bites. Spinal cord H4R-mediated itch can be persistent, and antagonists for H4R attenuated itch in AD patients whereas antagonists for H1R and H2R are largely ineffective in AD and psoriasis.^20^
Proteases	Proteases perform as any enzyme about proteolysis, which are involved in diverse physiological reactions. It is believed proteases are extremely important substances in causing histamine-independent pruritus.^27^ Recent studies have demonstrated that proteases play a crucial role in itching attack by combining to GPCR called Proteases Activated Receptors (PARs), especially PAR2 and PAR4.^26^	
Other Peptides	*Bradykinin*	Bradykinin belongs to an active peptide of the kinin group of proteins. It is a potent inflammatory mediator and endothelium-dependent vasodilator, which contribute to the production of inflammatory reaction and the dilation of blood vessels.^26^ The receptors of Bradykinin comprise Receptor B1 (B1R) and Receptor B2 (B2R) belonging to the members of GPCR family.^26^ By combining with its receptors, bradykinin initiates and induces a variety of physiological and pathological reaction.^26^
Phospholipids metabolites	*Cannabioides*	Cannabinoids (CB) belong to the derivatives of arachidonic acid, the receptors of which contain CB1 receptor and CB2 receptor. CB1 receptor is distributed in the central nervous system, while CB2 receptor is distributed in the peripheral tissues.^27^ CBs, by binding to their receptors, could induce the release of 13-endorphins, further, to relieve pain and alleviate histamine induced pruritus.^27^ These results indicate that CB may be involved in the regulation of pain and pruritus.^26^ In mice, both receptors have been detected in sensory neurons, whereas in humans, only CB2R has been so localized.^15^ Activation of CBR in sensory neurons may decrease neuronal activity and modulate the axon-flare response.^15^ Tetrahydrocannabinol, a bioactive component of marijuana, blocked scratching behavior elicited by compound 48/80.^15^
	*Eicosanoids*	There are multiple subfamilies of eicosanoids, consisting of Leukotrienes (LTs), prostaglandins, resolvins, lipoxins, eoxins, and thromboxanes.^26^ LTs, most prominently, are important regulators in the modulation of pruritus, and LTB4 levels are significantly elevated in AD and psoriatic lesions, which were usually accompanied with pruritus.^26^
	*Platelet-Activating Factor (PAF)*	PAF has a variety of physiological and pathophysiological effects, which acts as an important mediator and activator in anaphylaxis, inflammation, platelet aggregation and degranulation, and leukocyte chemotaxis.^26^ Normally, PAF is produced in low quantities by various cells (e.g., platelets, neutrophils, macrophages, endothelial cells, and monocytes), but it emerges in larger quantities from inflammatory cells in response to specific stimulator.^26^
*Cytokine and Chemotactic Factor Induced Itch*	The cytokines build “a bridge of communication” between the immune system and the nervous system. AD-related skin lesions and itch are aggravated under the mutual interaction of neural–epidermal immune signal pathways.^20^ Pruritus is caused by a variety of pruritus-derived cytokines, including TSLP, Interleukin-2 (IL-2), Interleukin-4 (IL-4), Interleukin-13 (IL-13), IL-31, Interleukin-33 (IL-33), etc., and by the imbalance of the neuro-immune circuit between the receptors IL-4R, IL-13R, IL-31RA, OSMR, Mrgprs, and itching peptides (SP, BNP, CGRP, GRP and protease, etc.).^20^ As the quantity of TH2 cells is increased, the inflammation related to specific cytokines and the generation of eosinophilia and Immunoglobulin E (IgE) are promoted, whilst the generation of epidermal barrier proteins and antibacterial peptides is inhibited. IL-4 and IL-13 are typical type-2 cytokines and have been proven to directly stimulate the sensory neurons via the Janus Kinase-1 (JAK1) signals and promote itch sensation.^20^	
	*IL-13*	IL-13 levels are increased in skin and serum from AD patients, and IL-13 participates in the initiation of AD and itching. Together with IL-4, it aggravates epidermal barrier dysfunction by downregulation of the Filaggrin (FLG) and Involucrin (IVL) expression in the keratinocytes.^20^ The sensory neurons and keratinocytes express heterodimer receptor IL-4, receptor alpha/IL-13, receptor alpha-1 (IL-4Rα/IL-13Rα1), and IL-13 receptor alpha-2 (IL-13Rα2).^20^ IL-13 binds with IL-13Rα1 with low affinity, and when the heterodimer receptor consisting of IL-13Rα1 and IL-4Rα is formed, with high efficiency, the latter is a type-II receptor.^20^ IL-13 and TLR2 heterodimer agonists can upregulate the transcription of IL-13Rα2 in keratinocytes and sensory neurons, respectively, thereby promoting neurogenic inflammation and exacerbating AD and itch.^20^
	*IL-31*	IL-31 plays an important role in the induction of itch and inflammation in AD and chronic contact dermatitis in mice and humans.^20^ IL-31 stimulates itch-related neuronal subset NP3, a subpopulation also responsive to mast-cell-released 5-HT, Leukotriene C4 (LTC4), and S1p, and release BNP and SST. Moreover, IL-31 binds to its receptors on epidermal keratinocytes and immune cells (i.e., eosinophils) to induce skin barrier dysfunction and cutaneous inflammation.^20^
	*IL-33*	IL-33 is an effective amplifier of type-2 immune reaction and is an important target for dry skin pruritus and chronic pruritus of unknown origin (CPUO).^20^ IL-33 receptor ST2 (also named IL-33R) is expressed in DRGs, keratinocytes, immune cells, fibroblasts, and mast cells.^20^ Binding of IL-33 to keratinocytes contributes to the impeded filaggrin and claudin-1 protein expressions and functional damages to the skin barrier, and facilitation of immune regulation.^20^ IL-33 stimulates diverse cells including ILC2 (innate lymphoid cell type-2) and generates type-2 cytokines including IL-5 and IL-13.^20^
	*TSLP*	TSLP is a pro-allergic cytokine that is mainly released from keratinocytes and is the prime target in AD. TSLP drives TH2-mediated inflammation and enhances periostin release from keratinocytes, thereby promoting itch signaling, and this effect is susceptible to JAK2 inhibitor SD1008 and the STAT3 inhibitor niclosamide. Upon release from keratinocytes, TSLP activates various immune cells such as T-cells, dendritic cells, mast cells, and sensory neurons directly to evoke itch behaviors.^20^ The biological functions of TSLP require heterodimer formation between the TSLP Receptor (TSLPR) and interleukin-7 Receptor-alpha (IL-7Ra).^20^ TSLPR activation of primary afferent sensory neurons requires TRPA1 but not TRPV1.^20^ TSLP is also important for promoting wound-induced hair growth and regeneration in mice, which may be an issue that should be considered to use TSLP antagonists for pruritus accompanied by hair loss.^20^
	*Periostin*	Periostin plays critical roles in pathogenesis of skin fibrosis, lesional AD, psoriasis, allergic skin inflammation, and prurigo nodularis.^20^ Periostin is released from dermal keratinocytes and fibroblasts upon stimulation by TH2 cytokines IL-13 and IL-4, then activates integrin aVβ3 on a fraction of SST+/NPPB + sensory itch fibers.^20^ Meanwhile, periostin stimulates keratinocytes and immune cells to release various cytokines, including TH2 cytokines such as IL-31.^20^ MC903 and house dust mites promote periostin release via a JAK/STAT-mediated mechanism.^20^ Periostin also induces TSLP release in a periostinTSLP-TH2 cytokine–periostin feedback loop.^20^
	*Lipocalin-2 (LCN2)*	LCN2 is a central modulator of chronic itch via a STAT3-dependent mechanism in the spinal astrocytes.^20^ It is also released by neutrophils and keratinocytes.^20^ The serum level of LCN2 is associated with the severity of itch in patients with psoriasis.^20^
	*IL-2*	IL-2 is an itch inducer as well as an autocrine cytokine, and its single intradermal injection induces a long-time low-intensity local skin itch that lasts 48–72 h, as well as erythema in human AD patients and healthy subjects.^20^ IL-2 is released from keratinocytes and various immune cells, then activates histaminergic neurons. Moreover, it induces erythema and dermal T-cell infiltration.^20^
	*IL-6*	IL-6 is predominantly expressed in dendritic cells, keratinocytes, macrophages, and neurons.^20^ The dendritic-cell-derived IL-6 level is linked to AD. IL-6 facilitates production of IL-4 expression by CD4+ T-cells and their differentiation to TH2 cells.^20^
	*Oncostatin M (OSM)*	OSM is released by dermal T cells, macrophages, dendritic cells, neutrophils, and monocytes. Its receptor OSMR resides in sensory neurons expressing BNP; however, OSM does not activate sensory neuronal calcium entry, thus it is different from other pruritogens.^20^ OSM also potentiates histamine- and leukotriene-evoked itch behaviors.^20^

Histaminergic and nonhistaminergic sensory nerves constitute the two major pathways of pruritus.^18^ The histaminergic pathway transmits acute and chronic itch, as in cases of acute or chronic spontaneous urticaria, and is mediated by histamine secreted primarily by mast cells, basophils, and keratinocytes.^18^ Once released, histamine binds to H1 and H4 receptors on histaminergic nerves, activating TRPV1.^18^ Nonhistaminergic itch is elicited by nerves that express several receptors, activated by pruritogens other than histamine.^18^ These pruritogens are released by a variety of effector cells, including mast cells, granulocytes, macrophages, lymphocytes, Innate Lymphoid Cells type-2 (ILC2), keratinocytes, and neurons.^18^ Recent evidence also suggests that basophils can promote itch mediated by Immunoglobulin E (IgE), independent of mast cells.^18^

(v) Pruritus in the spinal cord

**Fig. 3:**
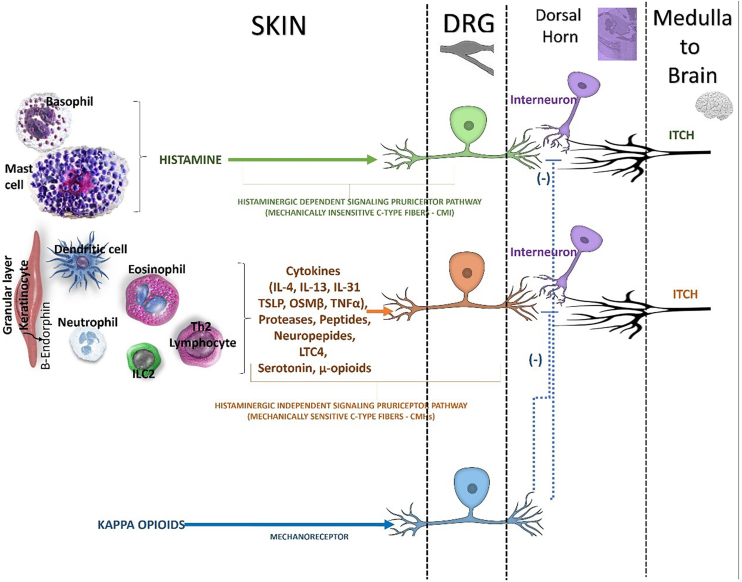
Pathways inducing and inhibiting the itch in spinal cord. Beside inflammatory mediators, it has long been appreciated that mu opioids (e.g, morphine) can trigger pruritus. Although it is well appreciated that mu opioids trigger itch, in part, within the central nervous system (CNS), mu opioid receptor (MOR) is abundantly expressed on pruriceptors fibers in the skin. Mu opioids act as direct pruritogens. The peripheral itch neurocircuitry have unveiled abroad array of nonhistaminergic pathways within the skin (epidermis and dermis), that can trigger various forms of itch. There are endogenous pathways dedicated to suppressing itch both in the periphery and the CNS. It is well known that mechanical stimuli (e.g., scratching) and pain may suppress the itch. This is likely due, in part, to inhibitory pathways being triggered within the spinal cord. In addition, endogenous kappa opioids, distinct from mu opioids, have been shown to suppress itch by their ability act on sensory neurons, the spinal cord (dorsal horn), and the brain. The activation of kappa opioid receptor (KOR) counterbalances the pruritogenic effect of MOR activation. However, KOR activation in the periphery is predominantly expressed on mechanosensory neurons (mechanoreceptors), rather than on pruriceptors. DRG, dorsal root ganglion; ILC2, innate lymphoid cell type 2; IL, interleukin, TSLP, thymic stromal lymphopoietin; OSMβ, oncostatin beta, LTC4, leukotriene C4; μ-opioids, mu-opioids; (-), inhibitory effect.

The itch signal is transmitted through the neuron cell bodies in the DRG to the dorsal horn of the spinal cord (Fig. 3).^18^ The activated sensory neurons release Gastrin-Releasing Peptide (GRP) which binds to GRP Receptor (GRPR)-positive intermediate neurons (interneurons) in the spinal cord.^18^ Structural abnormalities of the spinal cord can also modulate the itch signaling pathway, causing localized neuropathic pruritus.^18^ Specific populations of inhibitory interneurons were involved in the control of itch, and their dysfunction could lead to enhanced itch perception.^25^ Radiculopathy of cervical nerves a contribute to brachioradial pruritus, whereas in notalgia paresthetica the dorsal rami of intercostal nerves are involved.^18^

(vi) Itch in the brain (CNS)

After transmission through the spinal cord, itch signals running along the spinothalamic tract and reach the thalamus and parabrachial nucleus, followed by brain.^18^

Pruritus perception involves the primary and secondary somatosensory cortex, insula, and anterior cingulate cortex.^18^

Histaminergic and nonhistaminergic pruritus also activate distinct areas of the brain and can resemble pain perception. It is very important to remember that brain activation upon pruritic stimulation is different in patients with chronic itch compared to healthy volunteers. This has been shown in functional Magnetic Resonance Imaging (fRMI) studies for instance with atopic dermatitis patients, where the frontostriatal circuit is relevant for the itch. There are top-down inhibitory pathways from the brain stem modulating the itch signal at the spinal level.^18^ These pathways seem to be affected in patients with chronic itch.^26^

(vii) Efferent pathway of itch: motor action of scratching

In the CNS (brain), the main neurotransmitters are Noradrenaline (NA) and serotonin (5-HT).^19^

The population of NA + neurons is located in the *locus coeruleus*, while their α1 adrenergic receptors are found in inhibitory interneurons of the spinal cord. The 5-HT1A receptor, expressed in GRPR + interneurons, also plays a direct role in the efferent (descending) signaling of itch. The Periaqueductal Gray Matter (PAG) receives input from both the amygdala and the parabrachial nucleus, actively contributing to the central processing of the emotional component of itch.^27^ A subpopulation of Tachykinin-1-expressing glutamatergic neurons (TAC1+) promotes stimuli that sustain the itch-scratch vicious cycle. The anterior cingulate cortex forms part of a circuit with the anterolateral thalamic nucleus and dorsomedial striatum, modulating histaminergic itch via a spinal circuit dependent on Bhlhb5+ interneurons.^19^

## Pruritus classification and investigation according to the presence or absence of primary skin lesions

CP may continue apart of its cause and can acquire an independent disease status, with neuroimmune inflammatory behavior, in several condition.^13^ In Table 3, the current classification of chronic pruritus according to the International Forum for the Study of Itch (IFSI) is adapted and presented on their respective groups and categories.^28^

**Table 3:** Classification of chronic pruritus into groups and categories.^4^, ^28^

Clinical groups in chronic pruritus	Etiological categories
IFSI I: *chronic pruritus on primarily lesions (altered) skin (CPL)*: in the presence of a skin disorder (previously: pruritus cum materia). This type is related mainly in conditions related as category I	Category I: dermatological origin of chronic pruritus (related to inflammatory dermatoses, infectious dermatoses, autoimmune dermatoses, genodermatoses, dermatoses of pregnancy, neoplasms, etc.)
IFSI II: *chronic pruritus on primarily non-lesional (unaltered) skin (CPNL)*: without the initial presence of a skin lesions (previously: pruritus sine materia). Usually, the clinical aspect is a normal skin, and have as etiology related to mainly category II, III, IV. Some conditions classified in this class include: pruritic skin disease before skin eruptions, disorders of iron metabolism, uremia, hepatic disease (especially cholestasis), internal malignancy, hematological disorders, infections, endocrine disorder, neurological disorder, heart failure, somatoform conditions, adverse drug reactions, pregnancy, pruritus of elderly skin and pruritus of unknown origin (CPUO)	Category II: Systemic origin of chronic pruritus (related to endocrinological and metabolic disorders, infectious diseases, hematological and lymphoproliferative disorders, visceral neoplasms, pregnancy, drug-induced pruritus, etc.) Category III: neurogenic origin or neuropathic diseases. a) Neurogenic origin (without neuronal damage): few clinical examples yet, potentially hepatic itch with increased µ-opioids (dis-inhibition of itch) b) Neuropathic diseases (neuronal damage causes itch): multiple sclerosis, neoplasms, abscesses, cerebral or spinal infarcts, brachioradial pruritus, notalgia paresthetica, meralgia paresthetica, post-herpetic neuralgia, vulvodynia, small fibre neuropathy Category IV: somatoform pruritus (psychiatric/psychosomatic diseases, depression, anxiety disorders, obsessive-compulsive disorders, schizophrenia, tactile hallucinoses, fatigue Category V: mixed (chronic pruritus as result of more than one conditions classified on distinct categories; overlapping and coexistence of several diseases)
IFSI III: *chronic pruritus with severe scratch lesions*: predominance of chronic scratch lesions (for example, chronic prurigo, lichen simplex) precluding the classification into the first or second group. The clinical picture is related to chronic secondary scratch lesions like prurigo nodularis, and the etiology may relate to categories I-IV	
	Category VI: chronic pruritus of undetermined/unknown origin (CPUO)

Also, for the comprehension of pruritus, some important terminologies are important, such as: allodynia, which refers to pain or itching caused by stimuli that are typically non-painful or non-pruritic, and is associated with central sensitization; alloknesis, the triggering of itch from stimuli that usually do not provoke pruritus; atmoknesis, which is itch that occurs when the skin is exposed to air, such as when clothing is removed; central sensitization, describing the increased responsiveness of nociceptive neurons in the central nervous system to normal or subthreshold stimuli, often linked to peripheral injury or inflammation, resulting in heightened excitability of central pathways, reduced inhibitory activity, and the development of chronic pain or itch; dysesthesia, an abnormal sensation that may include burning, itching, pain, or tingling, often associated with the scalp; and neurogenic inflammation, which is related to the release of mediators, such as substance P (SP) or calcitonin gene-related peptide (CGRP), from peripheral afferent neurons, impacting the immune system.^29^

Outcome measures in pruritus were created to help clinicians and researchers evaluate the severity of pruritus in a standardized and quantifiable manner, reported by the patients. The main outcome measures include:

a) Visual Analog Scale (VAS): patients rate the intensity of pruritus on a continuous line, ranging from 0 (no itching) to 10 (worst imaginable itch);^30^
b) Numerical Rating Scale (NRS): similar to VAS, NRS involves patients assigning a numerical value (e.g., 0‒10) indicating the severity of pruritus. It provides a quick and straightforward assessment of itching;
c) Dermatology Life Quality Index (DLQI): represents the impact of pruritus on a patient´s quality of life, including questions from different domains, such as symptoms, daily activities and emotional well-being, providing a comprehensive view of how itching affects various aspects of life;^31^
d) Itch Numeric Rating Scale (Itch NRS): This is a specific numerical rating scale designed for assessing the severity of itch, ranginf from a value of 0‒10;^32^
e) Patient-Oriented Eczema Measure (POEM): Originally designed for eczema, this questionnaire evaluates the impact of pruritus on symptoms and its effect on daily activities.^33^

### Diagnostic workup in chronic itch

An essential step in addressing CP involves a meticulous recording of the patient's medical history and a thorough clinical examination (Table 4). Additionally, an interdisciplinary diagnostic workup, incorporating laboratory tests and imaging studies, is imperative for the diagnosis.^13^

i Chronic pruritus related to dermatological conditions

**Table 4:** Relevant medical data of in anamnesis of patients suffering from chronic pruritus.^28^

Characteristics of the itch		-date of the onset, duration
		-anatomical distribution: localization (onset, spread or localized)
		-quality (typification: pure pruritus, neuropathic characteristics)
		-intensity: (severity on numeric rating scale)
		-course: diurnal fluctuations, continuous/attack-like or paroxysmic course, spontaneous improvement/aggravation, nocturnal aggravation
		-provocation factors (example, aquagenic, physical exercise), alleviation factors (example, cold, skin covered by clothes)
		-scratching behavior
		-temporal association with pre-existing diseases, surgeries, intake of medications, other events
		-previous therapies with/without success
		-patient´s perception of the itch cause
		-factors of psychosocial burden
		-impairment of health-related quality of life, mental distress, sleep disturbances
General information		-pre-existing diseases, including dermatoses
		-previous surgeries
		-travel history
		-pregnancy
		-atopic conditions associated
		-allergies: type I and type IV
		-intake of medications, infusions, blood transfusions
		-living on endemic area of infestations
Screening questions related to anxiety and depression disorders		• Screening of depression:
		• In the last month, did you often feel low, melancholic, or hopeless?
		• In the last month, did you often have little interest or enjoyment in your activities?
		If both questions are answered in the negative, major depression can be excluded with a high sensitivity of 96%.
		• Screening of anxiety disorders:
		•During the last 4-weeks, did you feel significantly impaired by: nervous tension, anxiety, feeling to have lost your mental balance? Did you worry about all kinds of things?
		•During the last 4-weeks, did you have an anxiety attack (sudden feeling of anxiety or panic?)
		The anxiety screening has a sensitivity of 86% and a specificity of 83%.
Particular characteristics of medical history	Several family members are affected	Scabies or other parasitic diseases
	Pruritus after contact with water	• Aquagenic pruritus associated with lymphoproliferative diseases (example, polycythemia vera);
		• Pruritus during contact with water (showering, bathing) irrespective of the temperature or during cooling of the skin after bathing
	Pruritus during/after physical exercise	Cholinergic pruritus
	Pruritus and icterus	• Pancreatic cancer
		• Cholestatic hepatitis
		• Intrahepatic cholestasis of pregnancy
	Pruritus in winter	• Xerosis
		• Asteatotic eczema
	Pruritus with B symptoms (weight loss, drenching night sweats, fever, and generalized pruritus)	• Internal neoplasms
		• Lymphomas (such as Hodgkin´s disease, chronic lymphocytic leukemia)

**Fig. 4:**
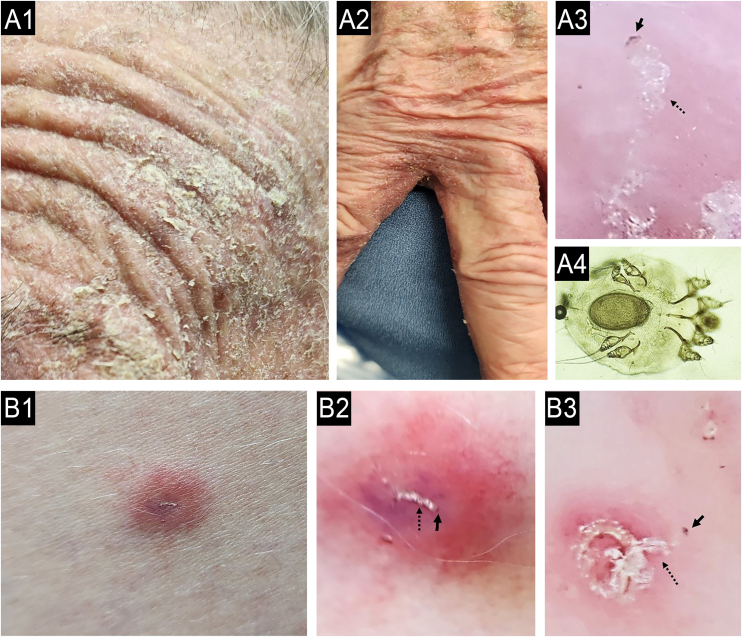
(A) Case of chronic pruritus misdiagnosed as drug adverse reactions (case A1-4) and other (B) as atopic dermatitis (case B1-3) presenting as scabies surrepticious, diagnosed by dermoscopy and direct scrapping of the skin guided by dermoscopy. A: A 87-years-old female patient suffering with chronic pruritus during 7 months noticed a history of 15 days of hospitalization due to a clinical hypothesis of drug adverse reaction; A1: erythematous = scaling lesions on face; A2:erythamtosus-descamative interdigital lesions on hands; A3: Dermoscopy of interdigital area showing “jet with contrail” (burrow´s acari) (dotted black arrow) and the “delta wing´s sign” (full black arrow) (30x magnification); A4: optical microscopy (KOH staining, 800x magnification) of the scales collect of the skin oriented by Dermoscopy examination, a female adult *Sarcoptes scabei var. hominis* showing an egg in the her body). (B) A female patient suffering due to chronic pruritus and scattered papules on trunk during the last 6 months, misdiagnosed as adult atopic dermatitis, treated with first three months with upadacitinib 30 mg/day, and after due to intractable itching, the physician associated dupilumab to treatment; B1: erythematous papules in lateral thigh; B2: dermoscopy showing the “jet with contrail” (dotted black arrow) and full black arrow demonstrating the “delta wing´s sign”, 10x magnification; B3: dermoscopy of plantar lesions showing “jet with contrail” (burrow´s acari) (dotted black arrow) and the “delta wing´s sign” (full black arrow) (400x magnification).

When a primary dermatosis is present, the differential diagnosis can be defined through a careful consideration of the clinical history and dermatologic examination.^34^ Unfortunately, CP has been globally misdiagnosed, primarily attributed to the incorrect diagnosis and treatment of patients with chronic scabies, especially in atypical presentations (Fig. 4). Therefore, dermoscopy examination and direct microscopic examination become critical for dermatologists.^35^, ^36^

Biopsy of skin dermatoses, including Hematoxyllin-Eosin stain (HE), immuno-histochemistry (especially in cases where cutaneous lymphoma is a differential diagnosis), and direct immunofluorescence (to rule out autoimmune dermatoses) may be extraordinarily helpful in this scenario if the etiology is not readily evident from history and physical exam alone.^34^ Some relevant laboratory exams (e.g. IgE serum levels, eosinophils count in peripheral blood, indirect immunofluorescence) are very useful for etiological diagnosis in chronic pruritus.^37^, ^38^, ^39^

ii Chronic pruritus secondary to internal or systemic diseases

### Iron deficiency and pruritus

Normally, it is present as generalized pruritus without a skin primary cutaneous lesion.^2^ The most attributable cause of generalized pruritus in patients with underlying systemic disease was found to be iron deficiency anemia, which responded to iron replacement.^2^ In all cases of Generalized Pruritus Without Rash (GPWOR), especially where iron loss is suspected, it is important to enquire about diet (vegetarian or vegan), potential sources of blood loss, previous bariatric surgery, and gastrointestinal symptoms. Iron replacement leads in some patients to complete cessation of pruritus very shortly after to introduction of iron therapy.^2^

When iron deficiency is suspected but ferritin levels appear to be within the 'normal' range, it may be necessary to check serum iron and total iron-binding capacity as well. A trial of iron replacement should be considered if ferritin levels are below the lower limit of the reference range (15 to 25 µg/L), or if there is anemia or microcytosis not attributable to other causes (e.g., gastrointestinal blood loss, urinary loss, thalassemia trait, or polycythemia). Individuals with unexplained iron deficiency should also be tested for Tissue Transglutaminase (TTG) antibodies, particularly anti-transglutaminase-2 antibodies, provided they have not been excluding gluten from their diet for at least six weeks. If the TTG test results are abnormal, referral to a gastroenterologist for consideration of endoscopy and small bowel biopsy is recommended. A biopsy may be indicated even with a negative TTG result, as IgA deficiency, which is relatively common, can lead to a falsely negative TTG measurement.^2^

### Iron overload

It may also be associated with generalized itch, either in association with hemochromatosis or hiperferritinemia in the absence of hemochromatosis.^2^

### Hematological and other malignant neoplastic causes related to chronic pruritus

A retrospective population level cohort study included 327,502 eligible patients diagnosed with unspecified itch with matched controls. Comprised 68.1% females, 59.3% white race, 22.2% black race, and a mean age of 42.2 ± 22-years. Pruritus patients had increased 1-year risk of Hodgkin’s lymphoma (RR = 4.42, 95% CI 2.83–6.88), myeloid leukemia (RR = 2.56, 95% CI 1.79–3.67), multiple myeloma (RR = 2.38, 95% CI 1.66–3.42) non-Hodgkin’s lymphoma (RR = 2.35, 95% CI 1.96–2.82), monoclonal gammopathy (RR = 1.90, 95% CI 1.55–2.32), myelodysplastic syndrome (RR = 1.74, 95% CI 1.14–2.64), and lymphocytic leukemia (RR = 1.47, 95% CI 1.07–2.02).^40^ The authors concluded that undifferentiated pruritus is highest in the first 12-months, and LDH (Lactate Dehydrogenase) has limited diagnostic utility in these patients. Providers should screen patients with undifferentiated pruritus for hematologic malignancies as clinically indicated.^40^ In patients with chronic pruritus without concomitant dermatologic diagnoses, older age, male sex, liver disease, and tobacco abuse increase the odds of an underlying malignancy.^41^

Itch can be a prodrome of malignancy, often appearing before other signs and symptoms. It is particularly common in hematologic malignancies, with prevalence estimates of up to 30% in patients with Hodgkin lymphoma, 15% in those with non-Hodgkin lymphoma, and 67% in patients with polycythemia vera. Lymphoproliferative disorders likely involve the expression of Th2-related cytokines, including IL-3 and TSLP.^39^

Patients with polycythemia vera often experience aquagenic pruritus, triggered by contact with water of any temperature, typically within minutes of exposure, and often more severe with hot water. Other hematologic conditions can also present with generalized pruritus, which may be accompanied by eczematous, urticarial, or lichenified skin findings, such as in hypereosinophilic syndrome. Among solid tumors, there is a notable association between pruritus and cancers of the hepatobiliary system. Although solid malignant tumors are a relatively rare cause of pruritus, Table 5 highlights malignant neoplasms associated with CP and their clinical characteristics.^39^

**Table 5:** Clinical characteristics (symptoms and signs) of some cancer associated with chronic pruritus.

Malignant neoplasms associated with chronic pruritus	Symptoms (1) and Signs (2)
All types of cancers (including hematological neoplasms)	(1) Loss of appetite, lethargy
	(2) Weight loss, lymphadenopathy, fever
Breast cancer	(1) Breast or axillary lump, change in breast shape, bloodstained nipple discharge
	(2) Breast or axillary lump, change in breast shape, bloodstained nipple discharge
Cholangiocarcinoma	(1) Nonspecific upper abdominal discomfort
	(2) Jaundice, pale stools, dark urine
Colorectal cancer	(1) Persistent change in bowel habit, diarrhea, abdominal pain, discomfort or bloating brought on by eating
	(2) Blood in the motions, in the absence of hemorrhoids on examination
Gastric cancer	(1) Persistent nausea, reflux symptoms, dysphagia or vomiting
	(2) Melaena, jaundice; very rarely, cutaneous stigmata of acanthosis nigricans and/or tripe palms
Gastric carcinoid tumour	(1) Abdominal pain, diarrhea, intermittent facial and/or trunk flushing
	(2) Very rarely, cardiac valve murmurs, cutaneous stigmata of neurofibromatosis type 1 or tuberous sclerosis
Insulinoma	(1) Intermittent double vision or blurred vision, confusion, anxiety and irritability, dizziness, mood swings, weakness, sweating and hunger
	(2) Symptoms correlate with episodic hypoglycemia
Lung cancer	(1) Persistent cough and breathlessness, persistent chest or shoulder pain
	(2) Persistent chest infections and wheeze, facial swelling, hoarse voice, finger clubbing. Very rarely, cutaneous stigmata of acanthosis nigricans and/or tripe palms, or dermatomyositis
Testicular cancer	(1) Intermittent dull ache or sharp pain in the testicle or scrotum
	(2) Clinical difference between one testicle and the other in texture or firmness.
Thymoma	(1) Persistent cough, shortness of breath, pain or pressure in the chest, diplopia, dysphagia
	(2) Anemia, frequent infections, muscle weakness, ptosis, arm or facial swelling. Very rarely, cutaneous stigmata of paraneoplastic pemphigus or pemphigus vulgaris/foliaceous

Generalized pruritus in malignancy is usually multifactorial.^2^ It can be a true paraneoplastic symptom, a feature of paraneoplastic dermatoses, secondary to paraneoplastic neuropathy, a consequence of secondary skin involvement by cutaneous or noncutaneous primary tumors, or a side-effect of cancer treatment.^2^ Paraneoplastic itch is defined as an itch that arises early in the course of malignancy or even precedes its clinical diagnosis. It is not caused by the invasion or compression of the neoplastic mass and typically resolves after the tumor is removed.^42^ Paraneoplastic skin diseases associated with itch of varying intensity can be classified into two groups: (i) Paraneoplastic syndromes, which include erythroderma, Bazex syndrome, Grover’s disease, the sign of Lesser-Trélat, generalized granuloma annulare, dermatomyositis, and malignant acanthosis nigricans, and (ii) Associated malignancies, which encompass hematological malignancies, and cancers of the head and neck, upper airway, digestive tract, colon, breast, ovaries, and nasopharynx.^43^ Although pruritus is thought to be an uncommon symptom in other solid malignancies, there have been case reports of itch occurring in patients with non-small-cell lung carcinoma, insulinoma, gastric carcinoid tumors, and other solid malignancies.^39^ Itching and burning sensation is reported among patients with glucagonoma syndrome. Patients with chronic unexplained pruritus that favors a possibility of underlying malignancy include older age, male sex, possible liver disease, and chronic tobacco usage.^2^ Also, several cancer treatments, including radiotherapy, can lead to pruritus by a variety of mechanisms.^2^

Paraneoplastic pruritus should be especially considered when chronic pruritus lasts less than 12-months.^2^ Many cancer treatments, including radiotherapy, can lead to pruritus by a variety of mechanisms. Treating the underlying malignancy can often alleviate pruritus. When cancer-drug-induced pruritus occurs, it may require modifying or discontinuing the offending medication. And biological therapies are now commonly used in oncology.^2^ A recent meta-analysis of 33 RCTs concluded that pruritus was a significant side-effect of cancer treatment with this class of agent.^44^ Pruritus is a common side effect of epidermal growth factor inhibitors, which have either biological or intracellular mechanisms of action.^2^ Oncology patients receiving biological therapies or chemotherapy (can cause itch by distinct mechanisms, for instance by inducing a small-fibre neuropathy) should be asked about pruritus on review.^2^

### Chronic pruritus related to renal disorders

Pruritus is a common symptom in advanced chronic kidney disease, affecting 40%–90% of hemodialysis patients. The itch associated with chronic kidney disease is linked to uremic xerosis and/or neuropathy, systemic inflammation, and an imbalance in the opioid receptor system, characterized by increased μ-opioid receptor activity and decreased κ-opioid receptor activity. Secondary hyperparathyroidism due to chronic kidney disease has also been suggested as a potential cause of generalized pruritus, although the mechanism remains unclear. This hypothesis is supported by small cohort studies that observed an improvement in itch following parathyroidectomy.^19^, ^39^

### Chronic pruritus related to endocrine disorders

Itch is more prevalent in diabetic patients than in healthy controls, with rates of 26% compared to 15%. Pruritus in Diabetes Mellitus (DM) may result from the harmful effects of elevated glucose levels on cutaneous nerve fibers, often manifesting as a consequence of diabetic polyneuropathy, particularly small-fiber neuropathy. Other endocrine disorders that can trigger pruritus include hyperthyroidism and hypothyroidism.^39^

### Chronic pruritus related to hepatobiliary disorders

Cholestasis from hepatobiliary conditions is a common cause of pruritus. These conditions include both primary and secondary causes of biliary obstruction, such as primary biliary cholangitis, primary sclerosing cholangitis, autoimmune hepatitis, intrahepatic cholestasis of pregnancy, viral hepatitis, and cirrhosis. Cholestatic itch arises from a complex interplay of factors, including bile acids, lysophosphatidic acid, bilirubin, and increased μ-opioid receptor activity.^39^ Cholestatic pruritus is often characterized by itch that initially affects the palms and soles, becoming more generalized as the disease progresses. The mechanisms behind HCV-associated pruritus are believed to involve HCV-induced cholestasis and the induction of interferon-stimulated genes due to viral overload.^20^

In the pediatric population, there are some distinct etiologies associated to chronic itch: primary sclerosing cholangitis (itch in 30% of these patients), biliary atresia, Alagille syndrome (45% of patients have itch), progressive familial intrahepatic cholestasis (itch in 76%‒100%) and benign recurrent intrahepatic cholestasis.^39^

### Other systemic etiologies for chronic pruritus including infestations and infections

Although their exact pathophysiology remains to be fully elucidated, other potential etiologies of itch may include exposure to heavy metals, vitamin deficiencies, HIV, and other viral infections. Elevated blood levels of heavy metals, such as cadmium and lead, have been associated with chronic itch. Additionally, low levels of vitamin D have been observed in patients with chronic pruritic skin conditions, including atopic dermatitis, psoriasis, and chronic urticaria. Low levels of vitamin B12 have been noted in patients with generalized itch from various systemic causes.^39^

Pruritus is commonly reported in patients with viral infections, particularly those living with HIV, where it correlates directly with viral load and can be associated with eosinophilia and eosinophilic folliculitis. Chronic itch is a significant comorbidity among HIV-positive patients, affecting 13%–45% of this population.^39^ Many HIV-positive patients also experience concurrent pruritic disorders, including lichen simplex chronicus, prurigo nodularis, scabies, seborrheic dermatitis, mycosis fungoides, and psoriasis. Additionally, itch in these patients may be caused by xerosis, drug therapies, and photosensitivity.^2^

Eosinophilia and generalized pruritus are features of parasitic infections, notably helminths such as *Strongyloides stercoralis,* but also onchocerciasis, cercariae dermatitis (due to skin penetration by cercariae of schistosomes or *Trichobilharzia* spp. in Western Europe).^2^ In tropical areas, pruritus may be a feature of arboviruses such as dengue, zika and less frequently, chikungunya infection.^2^, ^45^

A French prospective study followed 95 patients with pruritus *sine materia* over a period of five years (1996‒2001). In 40% of cases (38 patients), a systemic cause was identified. The main conditions included toxocariasis (8 cases), hematologic diseases (7 cases), chronic renal failure (6 cases), hypothyroidism (5 cases), and iron deficiency (5 cases). Neoplasms were found in eight cases (8.42%): seven involved hematologic malignancies (3 myeloma, 2 Hodgkin's disease, 2 myeloproliferative syndromes) and one involved a solid tumor (pulmonary adenocarcinoma). Toxocariasis, an often-underestimated disease, was the most frequently identified condition.^46^

Human toxocariasis is a parasitic disease characterized by the presence of larvae of the genus *Toxocara* in human tissues.^47^ *T. canis* and *T. cati*, found in dog and cat intestines, respectively, are the most common causative agents of the disease.^47^ Toxocaral larvae usually cause two severe syndromes: visceral larva migrans and ocular larva migrans, depending on the location of the larvae.^47^

iii Chronic pruritus related to neurogenic, neuropathic or central nervous system conditions

### Neuropathic pruritus

Neuropathic pruritic conditions may originate from the peripheral nervous system or central nervous system.^21^

The conditions originating from the peripheral nervous system are:^21^

a) Small fiber neuropathy: metabolic, drug-induced, infectious, or genetic origin (itch starts usually distally and may generalize);
b) Scars and burns: iatrogenic or traumatic (itch on lesional skin);
c) Radiculopathies: compression of a peripheral nerve by degenerative alterations or space-occupying lesions (itch and dysesthesias at the affected dermatome);
d) Postherpetic neuralgia: damage of peripheral nerve by the varicella-zoster virus (itch and dysesthesias at the affected dermatome);
e) Trigeminal trophic syndrome: injury of the sensory fibers of the trigeminal nerve (unilateral dysesthesia and hypoesthesia of the central face. Self-induced ulceration of the nasal ala, cheek, and upper lip).

The conditions originating from the central nervous system are:^21^

a) Space-occupying lesions: tumors, abscesses, vascular lesions, syringomyelia (clinical features according to affected neural structures);
b) Stroke: Ischemic or hemorrhagic (generalized or unilateral itch);
c) Multiple sclerosis: demyelinating disease (generalized itch or localized at the head and upper back);
d) Neuromyelitis optica: demyelinating disease (depending on injured spinal level);
e) Infectious diseases: meningitis, encephalitis, prion disease;
f) Traumatic brain or spinal cord lesions: accidents or iatrogenic lesions.

Generally, includes brachioradial pruritus, notalgia paresthetica, meralgia paresthetica, scalp pruritus (excluding dermatological diseases), gonalgia paresthetica (saphenous nerve damage), anogenital pruritus, and other conditions.^48^ The latter is caused by direct damage to the nerve itself.^48^ Although specific itch conditions have predominant contributors to their pathogenesis, it is most likely that there are multiple etiologies.^48^

Neuropathic pruritus refers to a group of disorders characterized by chronic itching caused by dysfunction or damage to pruriceptors.^48^ In these conditions, pruritus is not triggered by external stimuli, such as irritants or allergens, but rather emerges spontaneously.^20^ The neuropathic itch can occur owing to nerve damage that may be caused by mechanical, metabolic, inflammatory, or cytopathic injury.^48^

Pruritus neural hypersensitivity is exhibited in the following common neuropathic itch conditions.^20^ Brachioradial pruritus is characterized by itching on the arms bilaterally. It is often associated with compression or irritation of the nerves of the cervical spine.^20^ Notalgia paresthetica is a common chronic itch condition characterized by localized itching or burning sensation in the subscapular region and it may be caused by thoracic nerve damage or irritation in the affected area.^20^ Postherpetic pruritus is a complication of herpes zoster due to nerve damage caused by viral cytopathic changes.^20^ Finally, scalp pruritus is neuropathic when it occurs independently of any observable cutaneous eruption (e.g., seborrheic dermatitis).^20^ The damage to the occipital nerves from the cervical spine causes scalp pruritus.^20^

Painless self-injury from neuropathic itch is far more common on the face than anywhere else on the body.^49^ For example, itch is far more common after zoster affecting the face than the torso.^49^ The face is also unclothed and readily accessible to the fingers.^49^

Nerve fiber compression can cause pruritus in the corresponding dermatome, and nerve fiber degeneration (such as small fibre neuropathy) can cause localized or generalized pruritus.^4^ Small fiber neuropathy can occur in systemic diseases such as diabetes mellitus, Guillain-Barre syndrome, sarcoidosis, neurofibromatosis type 1 and HIV.^2^ Diabetic neuropathy can lead to a regional pruritus affecting the trunk.^2^ Small fiber neuropathy may be too small to produce clinical or electrophysiological changes, and the only investigation that may reveal anything is a skin biopsy with immunohistochemical staining of cutaneous nerve fibers.^2^

### Central nervous system, pruritus and delusional infestation

Central nervous system lesions affecting sensory pathways, such as strokes, multiple sclerosis, and cavernous hemangiomas, can lead to central itch. Damage to itch-transducing, conducting, or processing neurons can result in neuropathic pruritus. There are reports of patients developing new self-inflicted injuries decades after strokes or trigeminal surgery, often exacerbated by dementia, which causes uncontrolled scratching. Less common causes of central itch include multiple sclerosis, brain tumors, abscesses, and Sjögren’s syndrome. Rare cases have also been linked to anterior circulation strokes, particularly those affecting the thalamus.^49^

### Neurogenic pruritus

Neurogenic is a more general term that encompasses a pathologic process arising from the nerve.^20^ In other words, tumors of the nerve or other pathologies that have nothing to do with sensation or afferent transmission can still be referred to as neurogenic.^20^

Sensory nerves can contribute to neuroinflammation by releasing neuropeptides, which inflame tissues through efferent pathways. There are two primary ways in which sensory neurons can cause pruritus: (i) Neuropathic itch, where neuropathology leads to excessive afferent itch transmission to the CNS, and (ii) Neuroinflammatory processes, where sensory neurons activate immune cells or other intermediaries to trigger itch. A clear example of this is the release of substance P by sensory neurons, which binds to Mas-related G-protein-coupled receptor member X2 on mast cells, leading to the release of pruritogenic factors such as histamine and LTC4 from mast cells.^20^

A prime example of the neurogenic itch is Chronic Inducible Urticaria (CIndU). In this condition, various neurologic triggers, including thermal stimuli (heat, cold), mechanical stimuli (friction, pressure, vibration), and autonomic stimuli (acetylcholine), lead to the formation of hives and associated itch. CIndU exemplifies neurogenic itch, where itch is initiated by the nervous system in the absence of clinically defined neuropathic itch, likely through the activation of intermediate mast cells. Additionally, it is widely believed that Prurigo Nodularis (PN) also involves underlying neurogenic itch processes that trigger the development of cutaneous nodules.^20^

iv Chronic pruritus secondary to somatoform (psychiatric/psychosomathic) disorders

Chronic generalized pruritus is commonly associated with various psychiatric disorders, including depression, anxiety disorders, obsessive-compulsive disorder, substance abuse, and delusional infestation. Somatoform pruritus is characterized by itch where psychological, psychiatric, and psychosomatic factors play a crucial role in the onset, intensity, exacerbation, or persistence of the condition.^50^

v Chronic pruritus of undetermined/unknown origin (CPUO)

Once both underlying pruritic skin disease and other secondary causes of pruritus have been excluded, an individual may be considered to have CPUO.^2^ The prevalence of pruritus of unknown cause in individuals with generalized pruritus ranges from 3.6% to 44.5%, with the highest prevalence among the elderly.^13^, ^51^

The initial clinical approach includes a detailed medical history and full physical examination (including total body skin examination, lymph nodes palpation, liver and spleen examination, lung and heart auscultation, abdomen and pelvis palpation).^51^ Initial investigation should not only blood samples, but also urinalysis, stool routine, and occult blood, as well as X-Ray chest (radiologist to report), ultrasonography abdomen, and skin biopsy for direct immunofluorescence.^52^ On Table 6, described the main laboratory exams and complementary diagnostic approaches for patients with chronic pruritus of unknown origin.

**Table 6:** Workup directed for patient suffering with chronic pruritus of unknown origin (CPUO).^28^

Laboratory tests for all patients	Complementary tests in case of primary or secondary skin lesions, if necessary	Pruritus during pregnancy	Other possible exams/tests	Imaging technique (Even if medical history, physical examination, and laboratory tests do not result in a specific clinical suspicion, a chest X-Ray and an abdominal ultrasound may be performed to look for evidence of a potential malignancy)	Interdisciplinary cooperations
Erythrocyte sedimentation rate (ESR) and C-Reactive Protein (CRP)	Bacteriological/ mycological swabs	In case of prominent skin findings: dermatological examination to rule out polymorphic eruption of pregnancy (PEP), gestational pemphigoid-	***In case of anal pruritus***: parasites, worm eggs, digital rectal examination, PSA	Chest X-Ray	Neurological and/or psychiatric findings
Complete blood count with differential, ferritin	Skin biopsy (histology, direct immunofluorescence, electron microscopy)		***In case of aquagenic and genital pruritus, pruritus of unknown origin:*** lactose/sorbitol intolerance test	Abdominal ultrasound (including retroperitoneal lymph nodes)	
Bilirubin, transaminases (GPT [ALT], GOT [AST]), gamma-glutamyl transpeptidase (GGT), alkaline phosphatase,	Detection of scabies mites	In the absence of prominent skin findings: basic laboratory tests (see above) plus bile acids (fasting)	***In case of blood count abnormalities (example, bicitopenia)/suspected lymphoproliferative diseases***: vitamin B12, folic acid, protein electrophoresis, immunofixation, peripheral blood immunophenotyping (proliferative panel), JAK2 status, bone marrow biopsy (if necessary) with (immuno-)cytology and histology	Lymph node ultrasound (cervical, supraclavicular, axillary, inguinal), puncture/extirpation (if necessary)	Cooperation with other physicians and specialists: general medicine, allergology, dermatology, internal medicine, (gastroenterology, hepatology, endocrinology, hematology, and medical oncology), urology, gynecology, and others
				Thyroid ultrasound	
Creatinine, urea, estimated glomerular filtration rate (egfr), K+, urinalysis (test strip)				Gastroscopy (with biopsy and Helicobacter pylor*i* test, if necessary), colonoscopy (with biopsy, if necessary)	
Blood glucose level (fasting)			***In case of iron deficiency/stool irregularities***: stool guaiac test	CT, MRI, MRCP, scintigraphy (if necessary), ERCP (if necessary), liver biopsy (if necessary)	
Lactate Dehydrogenase (LDH)			***In case of suspected hepatobiliary disease***: hepatitis serology (anti-HAV, HBsAg, anti-HBc, anti-HCV), bile Acids, Antimitochondrial Antibodies (AMA), Perinuclear Antineutrophil Cytoplasmic Antibodies (pANCA), Antinuclear Antibodies		
Thyroid-Stimulating Hormone (TSH)			(ANA), Anti-Smooth Muscle Antibodies (SMA), anti-Soluble Liver Antigen Antibodies (SLA), anti-Liver-Kidney Microsomal Antibodies (LKM), anti-tissue transglutaminase antibodies, alpha-fetoprotein (in case of liver cirrhosis/hepatic mass)		
Complete blood count with differential, ferritin					
			***In case of abnormal fasting glucose levels***: HbA1c, glucose-tolerance test		
			***In case of primary or secondary skin changes***: direct and indirect immunofluorescence, autoantibodies against dermal proteins (BP180, BP230, desmoglein)		
			***In case of suspected allergy***: total IgE, specific IgE (if necessary), prick testing, patch testing		
			***In case of suspected endocrine disorders***: parathyroid hormone, phosphate, Ca2+, fT3, fT4, 25-OH cholecalciferol, anti-TSH receptor antibodies (TRAb), anti-thyroid peroxidase antibodies (TPO-Ab)		
			In case of suspected HIV: HIV serology, syphilis serology (if necessary)		
			In case of suspected mastocytosis: tryptase levels		
			- In case of suspected neuroendocrine tumors: chromogranin A		
			- 24 h urine collection: porphyrins (porphyria), 5-hydroxyindoleacetic acid (neuroendocrine tumors), methylimidazole acetic acid (mastocytosis)		

However, in some cases, the underlying cause remains unclear, and is Called Pruritus of Unknown Cause (CPUO).^51^ As CPUO is a diagnosis of exclusion, patients suffering from it are re-examined periodically.^51^

**Fig. 5:**
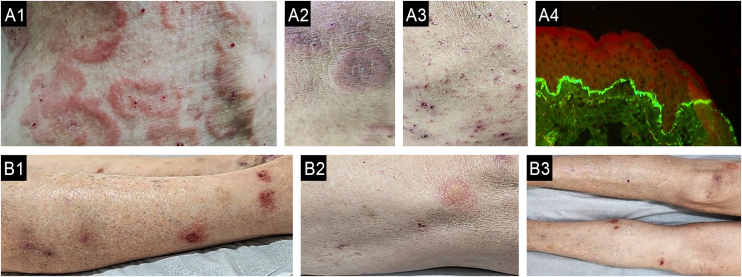
Unusual chronic pruritic conditions found on elderly patients: atypical bullous pemphigoid (ABP) (A) and eosinophilic dermatoses with hematological malignancy (B). Atypical lesions of bullous pemphigoid. A1: annular urticarial-like lesions with excoriated hematic crusted lesions; A2: urticarial plaques resembling urticarial vasculitis; A3: erythematous eczematous crusted lesions, with excoriated papules with hematic crusts and hyperchromic residual lesions; A4: direct immunofluorescence positive to IgG in a granular pattern on basal zone membrane of the skin, obtained from normal appearance skin of a 69-years-old female with 2 years complain of chronic itch. Eosinophilic dermatoses of hematological malignancy: image of a patient suffering from chronic itch for 2 years after covid-19, with progressive elevated count of lymphocytes on total blood count and a diagnosis of chronic lymphocytic leukemia. B1: showing erythematous excoriated and exulcerated lesions on ankles; B2: urticarial-like lesion resembling insect bites, and B3: several excoriated lesions on legs due to intense chronic itching.

CPUO may affect younger patients but is highly prevalent in those aged over 65 years.^2^ Although many factors are thought to underlie CPUO, physiologic changes associated with aging likely contribute, including epidermal barrier dysfunction, sensory neuropathy, immunosenescence, and aging are characterized by a proinflammatory state with enhanced type-2 immune responses and reduced type-1 immunity.^53^ In the elderly, besides the possibility of internal malignancy and cutaneous lymphoma, there are two chronic itchy conditions that should be remembered: atypical forms of bullous pemphigoid^54^, ^55^ and eosinophilic dermatoses with hematological malignancy (Fig. 5) (Table 8).^56^

vi Drug-induced chronic pruritus

**Table 7:** Drugs that may induce chronic pruritus.^34^

Antibiotics	Amoxicillin, ampicillin, cefotaxime, ceftazidime, clindamycin, ciprofloxacin, erythromycin, minocycline, metronidazole, penicillin G, rifampicin, tetracyclines, trimethoprim/sulfamethoxazole, vancomycin, antifungals, antimalarials
Antiuricosuric agents	Allopurinol, colchicine, probenecid, tiopronin
Cardiovascular agents	Amlodipine, amiodarone, candesartan, captopril, clonidine, diltiazem, enalapril, flecainide, irbesartan, lisinopril, methyldopa, verapamil
Hormones	Oral contraceptives, corticosteroids, clomiphene, danazol, estrogen, progesterone, testosterone, tamoxifen
Glucose-lowering agents	Metformin, glimepiride, gliclazide, gliptins, tolbutamide
Lipid-lowering agents	Clofibrate, fenofibrate, fluvastatin, lovastatin, pravastatin, simvastatin
Immunosuppressants	Cyclosporin, cyclophosphamide, metronidazole, mofetil mycophenolate, thalidomide, tacrolimus
Oncologic and biological agents	Adalimumab, 5-fluoracyl, cetuximab, chlorambucil, erlotinib, gefitinib, gemcitabine, infliximab, ipilimumab, nilotinib, panitumumab, paclitaxel, rituximab, temsirolimus, tamoxifen, vemurafenib
Opioids and analgesics	Acetylsalicylic acid, codeine, celecoxib, diclofenac, fentanyl, ketoprofen, morphine, naproxen, oxycodone, piroxicam
Neuroleptics, antiepileptics and antipsychotics agents	Amitriptyline, citalopram, chlorpromazine, carbamazepine, haloperidol, fluoxetine, paroxetine, phenytoin, risperidone, sertraline, topiramate
Miscellaneous	Antithyroid agents, iodine contrast, enoxaparin, interleukin-2, hydroxyethyl starch (HES), pentoxifylline, ticlopidine

**Table 8:** Special conditions associated with itch in the elderly patients.^46^, ^47^, ^48^

Skin condition	Distinct characteristics
Atypical bullous pemphigoid (ABP)	Up to 20% of the BP cases the onset is characterized by a non-bullous phase.^47^ Atypical manifestations of BP as described as:^47^ Non-bullous BP, annular-like; erythema multiformis-like; pemphigoid nodularis; lichen planus pemphigoids; exfoliative erythrodermic; vegetans BP; pretibial BP, and drug-induced or post-irradiation (PUVA, narrow band UVB therapy, photodynamic therapy or radiotherapy) BP.
	Nevertheless, all cases of atypical BP have the same immunopathological features of the classic form.^46^ The histological and Direct Immunofluorescence (DIF) findings and the detection of circulating autoantibodies, by Indirect Immunofluorescence (IIF), Enzyme-Linked Immunosorbent Assay (ELISA) or immunoblotting are the key elements to the correct diagnosis.^46^, ^47^ DIF of perilesional skin shows a continuous linear deposition of IgG (70%–90% of patients), or C3 (90%‒100% of patients), or both, along the Basement Membrane Zone (BMZ).^47^ Indirect Immunofluorescence (IIF) studies can demonstrate in 60%‒80% of patients the presence of circulating IgG autoantibodies that typically bind to the epidermal side of salt-split normal human skin.^47^ Circulating autoantibodies can be detected by Enzyme-Linked Immunosorbent Assay (ELISA), with sensitivity up to 100% when various ELISAs using the NC16A domain and other extracellular portions of BP180 or of BP230 are used together. Nowadays, ELISA has mostly replaced immunoblot and immunoprecipitation techniques, which are valid techniques but not very easy for routine testing.
	Cozzani et al. suggested diagnostic criteria for atypical bullous pemphigoid:
	*Major criteria*:
	a) DIG of perilesional skin showing a continuous linear deposition of IgG, C3 or both;
	b) Detection of circulating antibodies against BP 180 and/or 230 by ELISA and/or IIF on salt plit skin (with deposition og IgG and C3 at the roof).
	*Minor criteria*:
	c) Pruritus (itch)
	d) Histopathological findings:
	Early phases: subepidermal clefts, eosinophilic spongiosis, and/or an infiltrate of eosinophils in the upper dermis lining the dermal-epidermal junction are detectable.
	Established lesions: subepidermal bullae, absence of acantholysis, superficial dermal infiltrate of eosinophils.
	Polymorphic clinical aspects: widespread large, tense serous or hemorrhagic bullae on pruritic erythematous, urticarial or apparently normal skin; erythematous or urticarial plaques; polycyclic, annular, figurate lesions; hyperkeratotic excoriated nodules; plane, purple, polygonal, pruritic papules; small vesicles; erythroderma; vegetative, crusted purulent lesions.
	*The definitive BP diagnosis requires*: 2 major criteria or 1 major criteria plus 2 minor criteria.
Eosinophilic dermatosis of hematologic malignancy (EDHM)	Skin lesions composed by tissue eosinophilia arising in the context of hematologic disease is known as eosinophilic dermatosis of hematologic malignancy. The most associated malignancy is Chronic Lymphocytic Leukemia (CLL), although it has also been reported in association with other B-cell hematologic malignancies (mantle cell lymphoma, acute lymphoblastic leukemia, large cell lymphoma, multiple myeloma), and with acute monocytic leukemia, myelofibrosis, and nonmalignant processes, such as HIV infection and congenital agammaglobulinemia.
	EDMH is a rare condition with a wide variety of clinical presentations, ranging from papules, erythematous nodules, or blisters that simulate arthropod bites, to the formation of true plaques of differing sizes. The clinical presentation takes the form of pruritic papules or urticarial-like nodules reminiscent of arthropod bites. Cases have also been reported involving true plaques of variable size and occasionally vesiculobullous lesions. Lesions usually affect exposed and unexposed areas of the skin and occur predominantly on the limbs. Skin symptoms recur in situations in which insect bites would be unlikely. Histopathological skin exam reveals the presence of abundant eosinophils the presence of an eosinophil-rich, perivascular and periadnexal, interstitial inflammatory lymphocytic infiltrate, which occasionally extends into the subcutaneous tissue. The lymphocytic infiltrate is predominantly composed of T-cells (CD3+, CD43+, CD45RO+); when B-cells are present, they form polyclonal aggregates. Usually, no leukemic cells are observed.
	The diagnostic criteria for EDHM includes:
	a) Pruritic papules, nodules and/or vesiculobullous eruption refractory to standard treatment (as antihistamines, topical corticosteroids for itching).
	b) Eosinophil-rich lymphohistiocytic infiltrate in the upper and deep dermis.
	c) Exclusion of other causes of tissue eosinophilia
	d) Prior or subsequent diagnosis of hematological malignancy

Approximately 5% of cases of pruritus are caused by drugs. Drug-induced chronic pruritus may occur with or without a rash.^2^ Proposed mechanisms of drug-induced pruritus include cholestasis, direct drug or metabolite deposition, alteration of neural signaling, photodermatoses, xerosis or cell stimulation (for example, codeine), however, most cases are idiopathic.^2^, ^19^ Opioid-induced pruritus is common and affects 2%‒10% of patients receiving oral, 10%‒50% intravenous and 20%‒100% epidural and intrathecal opioids.^2^ The common culprits of drug-induced pruritus include immune checkpoint inhibitors;^39^ agents targeting epidermal growth factor receptor, B-Raf proto-oncogene, cytotoxic T-lymphocyte-associated protein-4, and Programmed cell Death protein-1/Programmed cell Death-Ligand-1 (PD-1/PD-1L).^39^ Table 7 displays the most frequent drugs that may induce chronic pruritus.^19^

## Treatment

The therapy of CP is quite complex and should be directed to their cause when it is found. There are, in most of the patients, especially in the elderly, several possible causes in the same patient, complicating the identification of the origin of CP.^57^ A temporal relationship between a certain cause and the onset of the pruritus may provide important hints about its origin.^57^

Therapeutic options may be restricted in certain populations, such as children, pregnant women, and breastfeeding or in elderly patients due to eventual concomitant stage of life, comorbidities and co-medication.^57^ In elderly patients, particularly, *xerosis cutis* is present in most patients and can be effectively treated with emollients.^57^ Systemic therapies should be used with caution, especially in very young patients or elderly patients after weighing risks and benefits.^57^ Additionally, polypharmacy often observed in elderly patients can hamper the choice of available therapeutic options due to possible interactions with antipruritic drugs.^57^

### Supportive care for itching patients

The main attitudes include prescribing continuous use of moisturization agents, especially adequate for patients with atopic dermatitis and/or sensitive skin (Table 9).^58^

**Table 9:** General measures and topical treatments for pruritus.

General measures^50^		
-reducing exposition to high temperatures and low relative air humidity;		
-trimming the nails to prevent excoriations;		
-avoid long baths;		
-avoid sauna for patients with itch triggering by these factors;		
-alleviating measures (e.g., wearing light or non-synthetic fibre clothes);		
-avoid smoking, alcohol, caffein and other stimulants, spices, and stress;		
-showering is better than taking a bath, with warm water, for 10 minutes at most;		
-shower with detergent-free soap (Syndet), shower oils, or cleansing cream;		
-avoid perfumed products and irritative substances such as sodium lauryl sulfate;		
-avoid bathing using antibacterial/antiseptic soaps;		
-emollient creams or hypoallergenic, free of fragrances and preservatives;		
-soft and loose-fitting cotton clothing.		

Topical Treatments		
Drugs	Mode of use	Observations
Hydrocortisone 1%^28^	1% 2×/day	Face and folds, or patients with thin skin
Betamethasone valerate 0.1%^28^	Topical or occlusive 1×/day	Extension areas
Tacrolimus 0.03%‒0.1%^28^	2×/day	Sensorial itching or a local burning sensation may present during the first applications and can be reduced with oral acetylsalicylic acid 500 mg, initially, and by avoiding alcohol consumption^34^
Pimecrolimus^28^	2×/day	
Capsaicin 0.025% to 0.1% cream^34^	4 to 6×/day	Induces TRPV1 activation with subsequent SP depletion, desensitizing the nerve fibers
Corticosteroids 19, 34	Occlusive or intralesional	Caution should be used due to the risks of skin atrophy and hypopigmentation.
Menthol 1% lotion^34^	3‒4×/day	Acts via TRPM8 pathway
Topical anesthetics:^34^		
-Polidocanol 2%‒10%		Application to localized (<10% of the body surface area, BSA
-Formulation: lidocaine 2.5%‒5% + amitriptyline 5% + ketamine 5%‒10% o/w cream	Transdermal application (3×/day)	Never more than 30% of the BSA)
-Pramoxine 1% lotion, cream, foam, gels	3‒4×/day	
		Cooling potentiates itch relief in neuropathic pruritus, via activaction of thermoreceptive type Aδ snd C nerve fiber^19^
-Lidocaine 2%‒5%	2‒4×/day	Take care with the occurrence of metahemoglobinemia if high doses of EMLA® (lidocaine + prilocaine) are applied in pediatric patients.
-Doxepine 5% cream^34^	4×/day 4/4h	There are risks of contact dermatitis, local sensation of itching/burning and anticholinergic effects (applied on <10% of the body surface area, BSA)
Botulinum neurotoxin	Intralesional 2‒10µ	Inhibits release of pruritogens, such as SP, CGRP, and acetycholine.^19^ Small studies have treated itch in lichen simplex chronicus and neuropathic etiologies of itch.^19^
Gabapentin 6%‒10%^19^	2‒4×/day	6% for uremic pruritus and 10% for scalp pruritus No drug-related adverse effects
Crisaborole1 19, 49	2×/day	A phosphodiesterase 4 inhibitor that reduces AD associated itch

Other alternatives		
Phototherapy UVA or UVB^49^		Efficacy in atopic dermatitis, cutaneous T-cell lymphoma and in CP arising from systemic diseases (e.g. end-stage renal disease, cholestasis). It may also be a viable treatment method for pruritus of unknown origin.

For all modalities of approach to treat CP, the experience has shown that it takes a long time before patients respond to treatment (up to 12-weeks); in case of cessation of itch, treatment should not be discontinued too quickly (gradual tapering over at least four weeks).^13^ The advent of Janus Kinase (JAK) inhibitors has ushered in a transformative paradigm shift, affording quick alleviation of pruritus.^59^

### Causal treatment

The range of causal treatments for pruritus includes addressing the underlying dermatosis, avoiding contact allergens, discontinuing medications, and employing specific systemic, neurological, and psychiatric therapies, as well as surgical interventions for tumors. In rare cases, treating or curing the underlying disease may lead to resolution of chronic pruritus. However, exceptions exist, such as short-term pruritus associated with Hodgkin’s disease and early chemotherapy, where the itch may not fully resolve even with treatment of the underlying condition.^13^

### Topical treatment

In CP basic therapy with emollients alone or in combination with specific topical, systemic agents and/or UV phototherapy is recommended.^13^ The first choice of topical treatment in lesional skin is topical corticosteroids, such as hydrocortisone (on the face or intertriginous areas),^60^ betamethasone valerate, or calcineurin inhibitors (do not combine with UV phototherapy).^13^ Calcineurin inhibitors may have a particular use in thin and sensitive skin areas, and prolonged use compared to topical corticosteroids.^61^

Another possible topical agent with antipruritic effect published only in case series or case reports is capsaicin which is indicated in neuropathic pruritus (notalgia paresthetica, brachioradial pruritus, postherpetic pruritus), aquagenic or uremic pruritus.^19^ In case of residual single nodules the use of intralesional corticosteroid intradermal injection is a possible alternative.^18^, ^19^

Localized CP may be treated with other agents, but the level of recommendation is based on case studies non-placebo controlled, such as menthol 1% lotion and topical anesthetics.topical antidepressive/histamine.^19^ intralesional botulinum neurotoxin, gabapentin 6%‒10%, phosphodiesterase inhibitors (crisaborole, difamilast, roflumilast).^18^, ^57^

### Phototherapy for chronic pruritus

Ultraviolet phototherapy [e.g., Narrow Band (NB) UVB 311 nm] constitutes an interesting option for all patients, including the elderly population as it is well tolerated, with few side effects and drug interactions.^19^, ^57^ Long treatment cycles lead to skin aging and can increase the risk of epithelial skin cancer.^54^

Excimer lamps also decrease the density of intraepithelial nerve fibers.^19^ UVA1 (340‒400 nm) decreases the levels of IL-4, IL-13, IL-17 and IL-23.^19^ Repeated exposure to suberythematogenic doses of both ofUVA1 and NB-UVB decrease IL-31 concentration, whereas high doses have the opposite effect, especially with UVB.^19^

### Systemic treatments

  a Antihistamines

Second-generation anti-H1 medications such as cetirizine, desloratadine, bilastine, rupatadine, fexofenadine, and levocetirizine are indicated in chronic urticaria. They cause less sleepiness than first-generation antihistamines and also interact with fewer medications. For the treatment of CPUO, the German Guideline for CP indicates as first line recommendation the second-generation H1 antihistamines (up to fourfold dosage).^13^ Most conditions involving CP have nonhistaminergic pathway involvement, then anti-H1 drugs have suboptimum effects or no effects to treat chronic pruritus.^13^

Oral first-generation H1 antihistamines, such as diphenhydramine and hydroxyzine, are commonly used as first-line options for CP, except in chronic urticaria, in children with milder cases, mostly due to their sedative properties.^58^ Side effects such as sedation and confusion from dialysis.^58^ The standard dosage for children is 5 mg/kg/d of oral diphenhydramine HCl or 2 mg/kg/d of hydroxyzine given at nighttime for sedation.^58^

  b Antidepressants agents^19^

These types of medications are indicated for uremic, cholestatic or paraneoplastic pruritus. The peak of the effect is reached after 4‒6-weeks. Adverse effects limit their use, particularly in the case of Selective Serotonin Receptor Inhibitors (SSRIs) and mirtazapine.^19^ These agents are less useful than gabapentin or pregabalin in neuropathic itch.^19^

Antidepressants should be used with caution in elderly patients due to reported severe side effects, mainly those of a cardiac nature.^57^ Serotonin reuptake inhibitors (e.g., paroxetine, fluvoxamine) can be used in somatoform, paraneoplastic, and aquagenic pruritus arising from hematological proliferative conditions.^57^ Sertraline has proven effective for the treatment of cholestatic pruritus.^57^ Tetracyclic antidepressants, namely, mirtazapine, amitriptyline, doxepin have also shown antipruritic effects on CP of various origins and beneficial effect on patients with impaired sleep quality due to CP (mirtazapine).^57^

Table 10 displays the main antidepressant medications used in distinct types of CP.

  c Gabapentinoids^19^

**Table 10:** Antidepressants used to treat chronic pruritus.

Antidepressant	Uses in distinct types of chronic pruritus	Adult dose	Children dose
Amitriptyline	Brachioradial pruritus, lichen amyloidosis, uremic pruritus, notalgia paresthetica, post-stroke pruritus, mycosis fungoides, HIV	25 mg/day	Not indicated
Doxepin	Uremic pruritus, pruritus of unknown origin	10‒20 mg/day	Starting dose 1 mg/day for infants, and maintenance doses range form 3 mg in an 8 kg baby with developmental delays to 10 mg in all children.^51^ The dose is escalated by 2 mg every 3-days to an average effective maintenance dose of 9 mg.^51^ Adverse effects in the form of behavioral side effects (aggression) and enuresis.^51^
Fluoxetine (SSRI)	Aquagenic pruritus	20 mg/day	Not reported
Fluvoxamine	Chronic pruritus, in patients with AD, lymphomas, paraneoplastic pruritus, psychiatric patients	25‒100 mg/day	Not reported
Paroxetine	Pruritus of unknown origin, psychogenic pruritus, paraneoplastic pruritus	20 mg/day	Not reported
Nortriptyline	Vulvar pruritus of unknown origin	74 mg/day	Not reported
Mirtazapine	Psychogenic pruritus, pruritus of unknown origin, paraneoplastic pruritus, nighttime pruritus of unknown origin	15‒30 mg/day (at night)	Children >10y-up to 15 mg orally; preferably at nighttime
Sertraline			1 mg/kg/day-4 mg/kg/day orally until maximum 100 mg/day

Withdrawal symptoms have been described with antidepressants as SSRIs (selective serotonin reuptake inhibitors) and SNRIs (serotonin-neuroepinephrine reuptake inhibitors) in children, adolescents, and adults over the past several decades and generally emerge when antidepressants are discontinued abruptly, although withdrawal symptoms can occur with missed doses and, in some patients, following significant dose reductions.^52^ These symptoms can include gastrointestinal (vomiting, diarrhea) and flu-like symptoms, dysesthesias, dyssomnia, increasing anxiety, agitation, or irritability and must be distinguished from recrudescence of symptoms associated with the disorder being treated.^52^

The general approach to managing antidepressant withdrawal is ensuring (i) adherence and (ii) slow discontinuation when stopping an antidepressant is necessary. For tapering in pediatric patients, using a typical taper, a patient taking sertraline 100 mg, then reduced to 50 mg, and then to 25 mg (i.e., 50% dose reduction at each titration point), every month.^52^

Gabapentinoids, especially gabapentin and pregabalin, are indicated for the treatment of neuropathic pain and can be also used to treat forms of neuropathy with both localized (e.g., brachioradial pruritus, notalgia/meralgia/gonalgia paresthetica, postherpetic neuralgia) and generalized (e.g., small fiber neuropathy due to diabetes) pruritus.^19^ Gabapentinoids have also shown efficacy in systemic diseases such as renal insufficiency and aquagenic pruritus.^19^ Depending on renal function, for adult patients gabapentin’s recommended dose is until 900 mg/day (however, higher doses as 3600 mg/day may be necessary) and pregabalin 75‒225 mg/day.^13^ In order to reduce the occurrence of side effects, it is important that the dose be slowly increased until therapeutic doses are reached.^19^ Additionally, the dose should be reduced in senior patients and those with impaired renal function.^19^ Common side effects include tiredness, dizziness, and weight gain.^19^ Gabapentinoids have few interactions with other drugs and thus constitute an appealing treatment option for older patients. The dosage of gabapentin in the elderly be initiated at 100–300 mg (pregabalin 25–75 mg) at night and slowly titrated up.^62^

  d Opioids

The Mu Opioid Receptor (MOR) antagonists and the Kappa Opioid Receptor (KOR) agonists have been shown to be very useful given their role as central pruritus regulators.^19^ These medications are classified as: (i) KOR agonists *difelikefalin* for parenteral use in cases of uremic pruritus in patients on hemodialysis; *nalfurafin* for parental use in patients with cholestatic pruritus, and *nalmefene* for oral use in cholestatic itch); (ii) KOR agonists/MOR antagonists: *nalbufin* for parenteral use in patients with uremic pruritus and prurigo nodularis; *butorphanol* for intranasal use and applied in intractable cholestatic itch; (iii) MOR antagonists: *naloxone* for parenteral use (reversal of side effects of opioids or intoxication) indicated for the drug-induced itch, brachioradial pruritus and cholestatic itch,^19^ and systemic administration of intravenous naloxone (0.002‒0.02 μg/kg body weight/min) provides rapid management of pruritus along with a low quantity of side effects;^62^ *naltrexone* for oral use (50‒150 mg/day), indicated for drug-induced itch, cholestatic itch, Hailey-Hailey disease, lichen planus pilaris, psoriasis, Darier´s disease.^19^ Low-dose naltrexone administered at 2 mg daily also has antipruritic effects and can be used in patients who cannot tolerate standard doses.^18^ MOR antagonist receptors, such as naloxone, naltrexone, or nalmefene, have been demonstrated to be effective in reducing itch in chronic urticaria, AD, PN as well as cholestatic and uremic itch.^62^ In contrast, activation of KORs inhibits pruritus.^57^ Butorphanol, a kappa-opioid agonist with some mu-opioid antagonist properties, has been shown in case series to effectively reduce itch due to PN, cholestasis, uremic itch, and idiopathic pruritus in elderly patients.^62^

The most common side effects of opioid modulators include gastrointestinal distress (such as diarrhea and vomiting), drowsiness, fatigue, dizziness, and insomnia. Additionally, there is a risk of liver injury at high doses, which necessitates caution when prescribing these medications to elderly patients.^18^, ^62^ Patients taking opioid agonists should avoid concurrent use of opioid antagonists, as this can trigger rapid withdrawal symptoms. Long-term use of agonists like butorphanol can also increase the risk of dependence. It is important to note that difelikefalin ‒ a selective kappa-opioid receptor agonist ‒ is FDA-approved only as an injection for the treatment of moderate-to-severe itching associated with chronic kidney disease in adults undergoing hemodialysis. Most other opioid medications used for chronic itch are used off-label.^18^

  e Bile acids resins and rifampicin

Cholestyramine is a bile acid resin that alleviates itch by sequestering pruritogenic bile acids. Rifampin, an antibiotic, helps reduce itch by promoting the conversion of bile acids to less pruritogenic forms. However, due to its hepatotoxic and nephrotoxic side effects, rifampin is not suitable for long-term use but can be an effective second-line treatment when cholestyramine is insufficient.^18^

  f Thalidomide

Thalidomide is a nonspecific immunomodulator that may disrupt the degeneration of type C unmyelinated nerve fibers.^65^ It can be effective in treating uremic pruritus and Prurigo Nodularis (PN). However, notable side effects include sedation, bowel obstruction, and peripheral neuropathy, which typically reverses upon discontinuation of the drug. Thalidomide is classified as a pregnancy category X drug due to its severe teratogenic effects, necessitating strict adherence to the Risk Evaluation and Mitigation Strategies (REMS) program. Thalidomide dosage is recommended in tablets of 50‒200 mg/day.^18^

  g Immunosuppressive drugs

Systemic immunosuppressive drugs are well-established in the treatment of inflammatory dermatoses, such as atopic dermatitis, psoriasis vulgaris, or cutaneous T-cell lymphoma.^57^ They should be considered following an unsuccessful treatment with topical or physical alternatives and after the exclusion of contraindications.^57^ Cyclosporine (2.5‒5 mg/kg/day), methotrexate (7.5‒20 mg weekly), mycophenolate (1‒2 g/day) and azathioprine (1‒3 mg/kg/day, often 50‒100 mg/day) are the most widely used substances.^18^, ^57^ Due to the complex interactions and undesirable side effect profiles of these drugs, they should be recommended with caution, particularly for older individuals. For instance, increases in retention parameters, liver enzymes, and hypertension are commonly observed. Therefore, a risk-benefit analysis should be conducted before initiating systemic immunosuppressive therapy, and patients should be thoroughly informed about the potential risks and side effects.^57^

  h Biologic therapies and oral small molecules

Dupilumab (600 mg initial, 300 mg Q2W), a monoclonal antibody targeting the receptor for IL-4, has been shown in large RCTs to reduce symptoms and improve quality of life in those with moderate to severe AD.^18^ The average age of participants in these trials was under 50, providing little evidence of efficacy in the elderly population.^18^ We have gained clinical experience using this drug in older patients with success, including an 87-year-old with itch and PN refractory to other treatments.^63^

Adverse effects have not been specifically detailed for the elderly, but in the general adult population, they include conjunctivitis, headache, and injection site reactions. There is no demonstrated increased risk of secondary infections, such as herpes viral infections or urinary tract infections, that would be of particular concern when prescribing to older patients.^18^ Several additional biologic therapies including targets of IL-31RA (nemolizumab for PN and AD), rocatinlimab (anti-OX40, in clinical trial for AD and PN), amlitelimab (anti-OX40 L, in clinical trial for AD) and lebrikizumab/tralokinumab (anti-IL-13, for AD) anti-OSMRβ (vixarelimab for PN) may control CP.^64^

Oral JAK inhibitors show promise in early phase trials for their antipruritic properties particularly, several itching dermatoses, including AD, PN, and chronic idiopathic pruritus).^18^, ^59^, ^63^ Baricitinib (JAK1/2-1), abrocitinib (JAK-1) and upadacitinib (JAK-1) are already approved for AD in several countries. JAK inhibitors such as tofacitinib (5‒10 mg/day) have adverse effects that should be strongly considered when used in the elderly including increased risk of herpes and other infections.^18^, ^65^, ^66^ A phase II clinical trial (NCT05038982) evaluating the efficacy of abrocitinib 200 mg during 12-weeks for reducing pruritus in adults with CPUO and prurigo nodularis was recently completed and is awaiting the publication of the results.^66^, ^67^

Based on the identification that JAK1 is expressed in neurons coupled with evidence of type 2 immune responses in CPUO, severe and refractory CPUO patients maybe could have benefits in the use of oral JAK inhibitors (not indicated to patients > 65-year-old in the atopic dermatitis setting).^53^ Dupilumab is currently being investigated in RTCs enrolling patients with CPUO.^68^, ^69^

## Conclusions

In conclusion, understanding the distinct categories of chronic pruritus, particularly the differentiation between pruritus with and without dermatological lesions is crucial for effective management. Pruritus without any dermatological lesions, often underdiagnosed or misdiagnosed, requires careful consideration and reevaluation. Recognizing these different forms of pruritus allows for a more targeted approach to treatment, addressing the specific pathophysiological mechanisms involved. Future research should continue to refine our understanding of these categories, potentially leading to more effective and personalized therapeutic strategies for patients suffering from chronic pruritus of various origins.

Ongoing research and development bring promise to the future of drugs targeting pruritus. Emerging pathways include compounds modulating neuroreceptors like Transient Receptor Potential (TRP) channels and opioid receptors.^70^ Advances in understanding molecular pathways open opportunities for developing targeted biologics and small molecules. JAK inhibitors, currently in use, might see refinement and broader application for pruritic conditions.

## Financial support

None declared.

## Authors’ contributions

Paulo Ricardo Criado: Design and planning of the study, drafting and editing of the manuscript and approval of the final version of the manuscript.

Roberta Fachini Jardim Criado: Design and planning of the study, drafting and editing of the manuscript and approval of the final version of the manuscript.

Mayra Ianhez: Design and planning of the study, drafting and editing of the manuscript and approval of the final version of the manuscript.

Hélio Amante Miot: Design and planning of the study, drafting and editing of the manuscript and approval of the final version of the manuscript.

## Conflicts of interest

Dr. Paulo Criado: *Advisory board -* Pfizer, Galderma, Takeda, Hypera, Novartis, Sanofi; *Pesquisa clínica* - Pfizer, Novartis, Sanofi, Amgen e Lilly; *Palestrantre:* Pfizer, Abbvie, Sanofi-Genzyme, Hypera, Takeda, Novartis.

Dra. Roberta Fachini Jardim Criado: *Advisory board ‒* Pfizer, Takeda, Hypera, Novartis, Sanofi; *Pesquisa clínica* ‒ Pfizer, Novartis, Sanofi, e Lilly; *Palestrantre:* Pfizer, Abbvie, Sanofi-Genzyme, Hypera, Takeda, Novartis.

Dra. Mayra Ianhez: *Advisory Board ‒* Galderma, Sanofi, Pfizer, Novartis, Abbvie, Janssen, UCB-Biopharma, Boehringer-Ingelheim; *Palestrante ‒* Galderma, Sanofi, Pfizer, Theraskin, Novartis, Abbvie, Janssen, Leopharma, FQM.

Dr. Hélio Miot: *Advisory Board* – Johnson & Johnson, L’Oréal, Theraskin, Sanofi e Pfizer; P*esquisa clínica ‒* Abbvie, Galderma e Merz.
